# Absence of KpsM (Slr0977) Impairs the Secretion of Extracellular Polymeric Substances (EPS) and Impacts Carbon Fluxes in *Synechocystis* sp. PCC 6803

**DOI:** 10.1128/mSphere.00003-21

**Published:** 2021-01-27

**Authors:** Marina Santos, Sara B. Pereira, Carlos Flores, Catarina Príncipe, Narciso Couto, Esther Karunakaran, Sara M. Cravo, Paulo Oliveira, Paula Tamagnini

**Affiliations:** a i3S-Instituto de Investigação e Inovação em Saúde, Universidade do Porto, Porto, Portugal; b IBMC-Instituto de Biologia Celular e Molecular, Universidade do Porto, Porto, Portugal; c Programa Doutoral em Biologia Molecular e Celular (MCbiology), Instituto de Ciências Biomédicas Abel Salazar (ICBAS), Universidade do Porto, Porto, Portugal; d Departamento de Biologia, Faculdade de Ciências, Universidade do Porto, Porto, Portugal; e Department of Chemical and Biological Engineering, University of Sheffield, Sheffield, United Kingdom; f Interdisciplinary Centre of Marine and Environmental Research (CIIMAR), Matosinhos, Portugal; g Laboratório de Química Orgânica, Departamento de Ciências Químicas, Faculdade de Farmácia, Universidade do Porto, Porto, Portugal; Aix-Marseille University

**Keywords:** *Synechocystis*, carbon fluxes, cyanobacteria, extracellular polymeric substances, polyhydroxybutyrate, secretion

## Abstract

Many cyanobacteria produce extracellular polymeric substances (EPS), composed mainly of heteropolysaccharides, that play a variety of physiological roles, being crucial for cell protection, motility, and biofilm formation. However, due to their complexity, the EPS biosynthetic pathways as well as their assembly and export mechanisms are still far from being fully understood. Here, we show that the absence of a putative EPS-related protein, KpsM (Slr0977), has a pleiotropic effect on *Synechocystis* sp. strain PCC 6803 physiology, with a strong impact on the export of EPS and carbon fluxes. The *kpsM* mutant exhibits a significant reduction of released polysaccharides and a smaller decrease of capsular polysaccharides, but it accumulates more polyhydroxybutyrate (PHB) than the wild type. In addition, this strain shows a light/cell density-dependent clumping phenotype and exhibits an altered protein secretion capacity. Furthermore, the most important structural component of pili, the protein PilA, was found to have a modified glycosylation pattern in the mutant compared to the wild type. Proteomic and transcriptomic analyses revealed significant changes in the mechanisms of energy production and conversion, namely, photosynthesis, oxidative phosphorylation, and carbon metabolism, in response to the inactivation of *slr0977*. Overall, this work shows for the first time that cells with impaired EPS secretion undergo transcriptomic and proteomic adjustments, highlighting the importance of EPS as a major carbon sink in cyanobacteria. The accumulation of PHB in cells of the mutant, without affecting significantly its fitness/growth rate, points to its possible use as a chassis for the production of compounds of interest.

**IMPORTANCE** Most cyanobacteria produce extracellular polymeric substances (EPS) that fulfill different biological roles depending on the strain/environmental conditions. The interest in the cyanobacterial EPS synthesis/export pathways has been increasing, not only to optimize EPS production but also to efficiently redirect carbon flux toward the production of other compounds, allowing the implementation of industrial systems based on cyanobacterial cell factories. Here, we show that a *Synechocystis kpsM* (*slr0977*) mutant secretes less EPS than the wild type, accumulating more carbon intracellularly, as polyhydroxybutyrate. Further characterization showed a light/cell density-dependent clumping phenotype, altered protein secretion, and modified glycosylation of PilA. The proteome and transcriptome of the mutant revealed significant changes, namely, in photosynthesis and carbon metabolism. Altogether, this work provides a comprehensive overview of the impact of *kpsM* disruption on *Synechocystis* physiology, highlighting the importance of EPS as a carbon sink and showing how cells adapt when their secretion is impaired, and the redirection of the carbon fluxes.

## Abstract (precis)

Most cyanobacteria produce extracellular polymeric substances (EPS) that fulfill different biological roles depending on the strain/environmental conditions. The interest in the cyanobacterial EPS synthesis/export pathways has been increasing, not only to optimize EPS production but also to efficiently redirect carbon flux toward the production of other compounds, allowing the implementation of industrial systems based on cyanobacterial cell factories.

## INTRODUCTION

Most cyanobacterial strains produce extracellular polymeric substances (EPS), composed mainly of polysaccharides, that can either remain associated with the cell surface (capsules, sheaths, or slime) or be released to the extracellular medium, referred to as released polysaccharides (RPS) (1). Over the last 2 decades, a variety of functions have been assigned to these EPS, namely, cell protection, adherence, formation of biofilms, sequestration of nutrients, and motility (2–7). Furthermore, the cyanobacterial extracellular polymers possess unique features compared to their bacterial counterparts, such as the diversity of constituent monomers (up to 13), including uronic acids (up to 2), amino sugars, and deoxysugars, and the presence of sulfate groups and peptides that may contribute to their biological activity (1, 8, 9). Overall, these characteristics make them attractive candidates for biotechnological/biomedical applications ranging from their use as gelling or emulsifying agents to their use in drug delivery and as therapeutic agents (10–14).

Consequently, there is increasing interest in understanding the cyanobacterial EPS biosynthetic pathways, not only to optimize production yields but also to engineer polymer variants tailored for a specific application (9). However, when the main purpose is to potentiate the use of cyanobacteria as “cell factories,” the highly energy-consuming process of EPS production can strongly impair productivity. Thus, comprehensive knowledge of their pathways is also required to efficiently redirect the carbon flux toward the production of other compounds of interest (15, 16).

The final steps of EPS assembly and export are mostly conserved throughout bacteria, following one of three major mechanisms: the ABC transporter-, the Wzy-, or the synthase-dependent pathway (17–19). The ABC transporter-dependent pathway translocates the fully polymerized polysaccharide to the periplasm using a two-protein complex, composed of the transport permease KpsM and the ATP binding component KpsT. The Wzy-dependent pathway relies on Wzx to translocate the oligosaccharide lipid-linked repeat units to the periplasm, where polymerization is performed by Wzy. For both pathways, export to the extracellular space occurs through the action of a polysaccharide copolymerase (KpsE and Wzc) and outer membrane polysaccharide export (KpsD and Wza) (17, 20–22). For the production of alginate, the synthase-dependent pathway requires a synthase, Alg8, to simultaneously polymerize and export the polymer across the plasma membrane to the periplasmic site (18, 23).

A cyanobacterial phylum-wide analysis disclosed the presence of genes encoding proteins from the three pathways (mainly from the first two ones) but often not the complete gene set that defines a single pathway (24). This complexity is also evident in the physical organization of EPS-related genes in cyanobacteria, with multiple copies scattered throughout the genomes, either isolated or in small clusters (8, 24), suggesting a more intricate mechanism for EPS production/regulation in cyanobacteria. Previous works, based mainly on the generation and characterization of knockout mutants of the model cyanobacterium *Synechocystis* sp. strain PCC 6803 (here *Synechocystis*), have confirmed the involvement of homologues of key proteins from both the ABC transporter- and Wzy-dependent pathways in cyanobacterial EPS production. Regarding the ABC transporter-dependent pathway, mutants in *Synechocystis slr0977* (*kpsM*), *sll0574* (*kpsM*), *slr0982* (*kpsT*), and *sll0575* (*kpsT*) produce EPS with monosaccharidic compositions different from that of the wild type (3). Slr0977 and Sll0574 have the Pfam domains typical of bacterial KpsM and Wzm, which are part of the EPS or O-antigen (OAg) ABC transporters, respectively (24). Due to the homology with Escherichia coli, *slr0977* (previously referred to as *rfbA*) was designated *wzm* (3). However, the absence of Slr0977 did not result in changes in the lipopolysaccharide (LPS) profile of *Synechocystis* (3), suggesting that this protein is a KpsM homologue. Regarding the mutants of putative Wzy-dependent components, *wza* (*sll1581*), *wzb* (*slr0328*), and *wzc* (*sll0923*) mutants were also shown to be involved in EPS production, exhibiting less capsular polysaccharide (CPS), less released polysaccharide (RPS), or less of both, respectively (25, 26). Until now, none of the generated mutants exhibited a substantial decrease in RPS production, and a *wzc:wzb* double mutant exhibited a decrease in CPS and an increase in RPS, suggesting that in the absence of the two proteins, RPS production is likely to be diverted to an alternative route (26). Altogether, these results support the involvement of different players and possibly cross talk between homologues of the different canonical bacterial pathways.

In this work, and in order to pursue the unraveling of cyanobacterial EPS assembly and export pathways, a *Synechocystis slr0977* (*kpsM*) knockout mutant was generated and extensively characterized. This gene was targeted, taking into account its relevant genomic location (within a cluster of genes related to sugar metabolism) and previous observations that the absence of *slr0977* leads to EPS with altered composition (3). The *kpsM* mutant was characterized in terms of growth/fitness, EPS production, protein secretion, and cell envelope ultrastructure. In addition, and to achieve a comprehensive overview, the transcriptomes and proteomes of the *kpsM* mutant and the wild type were assessed.

## RESULTS

**FIG 1:**
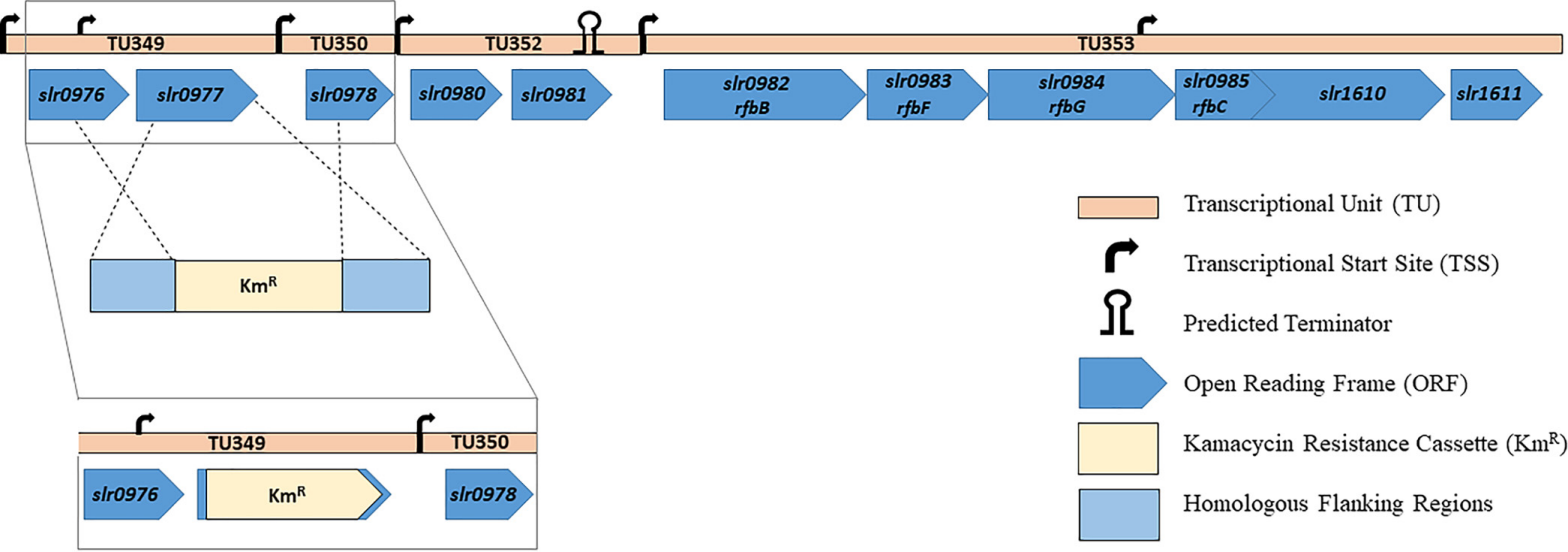
Schematic representation of the *slr0977* (*kpsM*) genomic context in *Synechocystis* sp. PCC 6803 and the generation of the *slr0977* knockout mutant by double homologous recombination. *slr0982*, *slr0983*, *slr0984*, and *slr0985* are annotated as *rfbBFG* and *rfbC*, while *slr1610* is annotated as encoding a methyltransferase (3). The remaining ORFs encode hypothetical proteins of unknown function. The transcriptional unit and transcription start site are annotated according to Kopf et al. (27). The predicted terminator was found using the FindTerm algorithm (Softberry); the insertion of the antibiotic cassette did not alter the prediction.

In the present work, an *slr0977* (*kpsM*) *Synechocystis* knockout mutant was generated and extensively characterized. This open reading frame (ORF) is located in a genomic locus containing 11 genes in which about half are putatively related to sugar metabolism (for details, see Fig. 1). The disruption of *kpsM*, which encodes a putative transport permease of the ABC transporter, was achieved via double homologous recombination by partially replacing the gene (749 bp out of 831 bp) with a kanamycin (Km) resistance cassette. The complete segregation of the mutant was confirmed by PCR and Southern blotting (see Fig. S1 in the supplemental material).

10.1128/mSphere.00003-21.2 FIG S1 Southern blot analysis confirming the segregation of the *Synechocystis* sp. PCC 6803 *kpsM* mutant. The DNA was digested with the endonuclease SpeI. A digoxigenin-labeled probe covering the 5′-flanking region of *kpsM* was used (primers are listed in Table S4 in the supplemental material). The sizes of the DNA fragments hybridizing with the probe are indicated. wt, wild type; #, clone tested. Download FIG S1, TIF file, 0.04 MB. Copyright © 2021 Santos et al. 2021 Santos et al. This content is distributed under the terms of the Creative Commons Attribution 4.0 International license.

### The *Synechocystis kpsM* mutant exhibits a light-dependent clumping phenotype and produces less EPS than the wild type.

**FIG 2:**
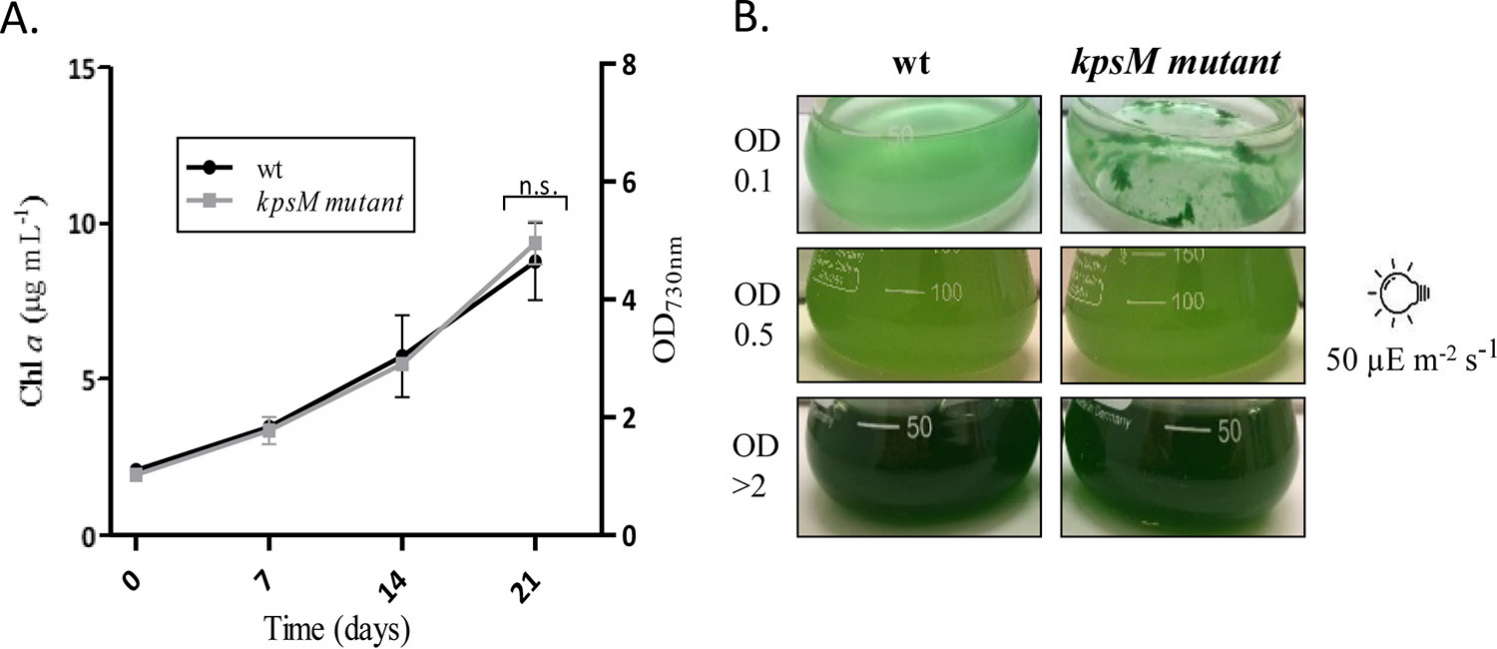
Growth curves of *Synechocystis* sp. PCC 6803 wild-type (wt) and *kpsM* mutant strains (A) and the respective phenotypes showing the clumping of the mutant cells at lower cell densities (B). Cells were grown in BG11 medium at 30°C under a 12-h light (50 μE m^−2^ s^−1^)/12-h dark regimen, with orbital shaking at 150 rpm. Growth experiments were performed in triplicate, and statistical analysis is presented for the last time point (n.s., not significant[*P* value of >0.05]). Chl *a*, chlorophyll *a*.

The *kpsM* mutant was initially characterized in terms of growth/fitness. Under the conditions tested (12 h of light at 50 μE m^−2^ s^−1^/12 h of dark, at 30°C and 150 rpm), the mutant strain did not show any significant growth differences compared to the wild type (Fig. 2A). However, at lower cell densities (optical density [OD] of <0.5), it displayed a clumping phenotype that faded with culture growth (Fig. 2B). Interestingly, this phenotype was not observed when cultures were grown at a lower light intensity (20 μE m^−2^ s^−1^) but was always visible for cultures grown at a higher light intensity (100 μE m^−2^ s^−1^). Gradual bleaching was also observed for the mutant cells exposed to 100 μE m^−2^ s^−1^ (data not shown).

**FIG 3:**
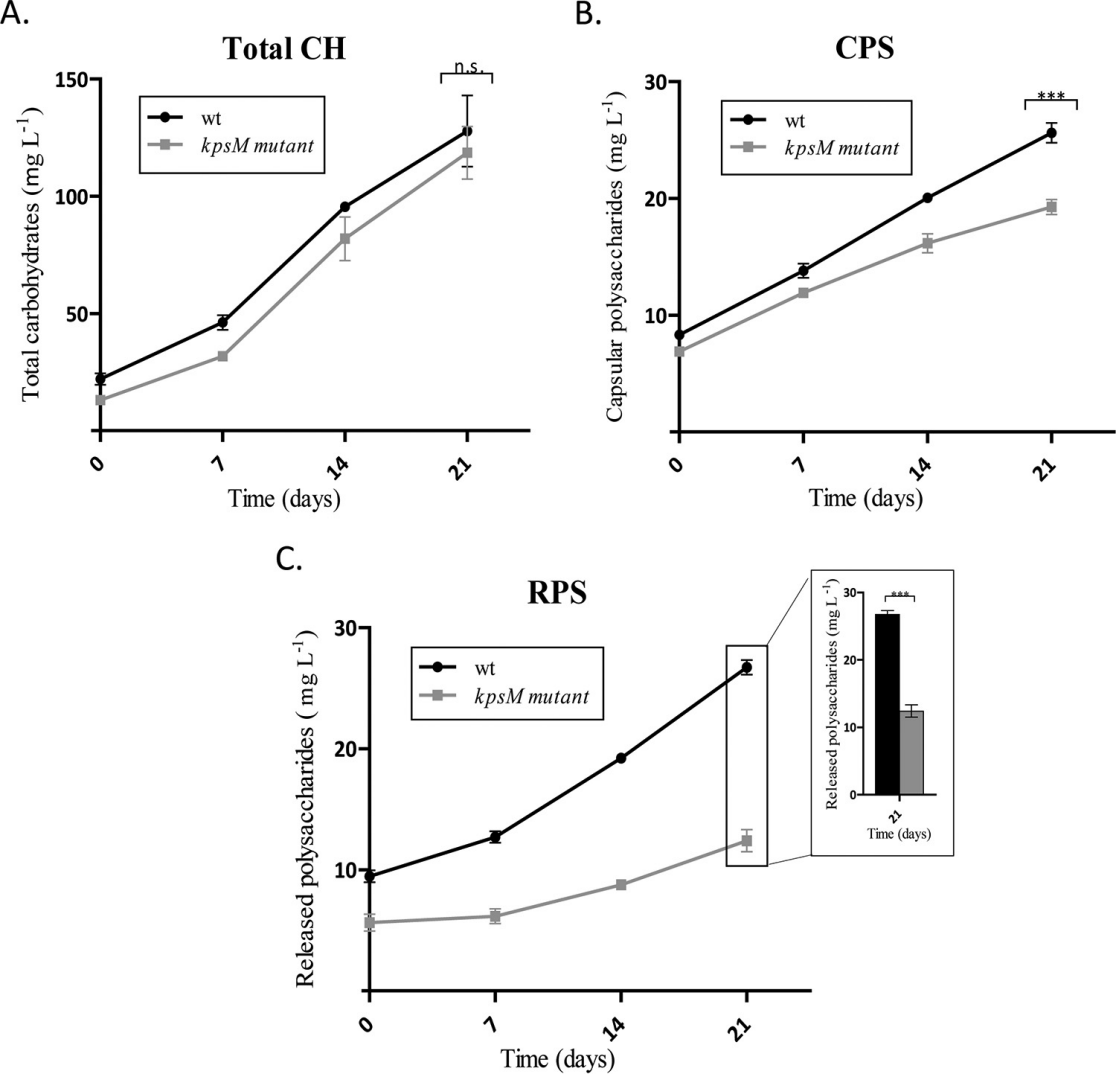
Total carbohydrates (Total CH) (A), capsular polysaccharides (CPS) (B), and released polysaccharides (RPS) (C) of *Synechocystis* sp. PCC 6803 wild-type (wt) and *kpsM* mutant strains, expressed as milligrams of carbohydrates per liter of culture. Cells were grown in BG11 medium at 30°C under a 12-h light (50 μE m^−2^ s^−1^)/12-h dark regimen, with orbital shaking at 150 rpm. Experiments were performed in triplicate, and statistical analysis is presented for the last time point (***, *P* value of ≤0.001).

The total carbohydrate contents were similar for the wild type and the *kpsM* mutant (Fig. 3A). However, the mutant showed 20% less capsular polysaccharide (CPS) (Fig. 3B) and released 50% less RPS than the wild type after 21 days of cultivation (Fig. 3C). Statistical analyses are presented for the last time point, as the differences accumulate with cell density (at this time point, the cultures no longer exhibit the initial clumping phenotype). The smaller amount of RPS secreted by the mutant, without a significant change in the total carbohydrate content, suggests a possible accumulation of carbon intracellularly.

To ensure that the observed phenotype was not due to polar effects, the knockout mutant was complemented in *trans* using the replicative vector pSEVA351 containing the native *kpsM* gene under the control of the psbA2* promoter. The complementation restored EPS production, reaching even higher levels of CPS and RPS than the wild type (Fig. S2).

10.1128/mSphere.00003-21.3 FIG S2 Total carbohydrates (Total CH), capsular polysaccharides (CPS), and released polysaccharides (RPS) of *Synechocystis* sp. PCC 6803 wild-type (wt) and *kpsM* mutant::pS351slr0977 strains expressed as micrograms of carbohydrates per microgram of chlorophyll *a* (chl a). Cells were grown in BG11 medium at 30°C under a 12-h light (50 μE m^−2^ s^−1^)/12-h dark regimen, with orbital shaking at 150 rpm. Experiments were performed in duplicate, and statistical analysis is presented (n.s., not significant [*P* value of >0.05]; *, *P* value of ≤0.05). Download FIG S2, TIF file, 0.04 MB. Copyright © 2021 Santos et al. 2021 Santos et al. This content is distributed under the terms of the Creative Commons Attribution 4.0 International license.

### The *kpsM* mutant accumulates more polyhydroxybutyrate.

**FIG 4:**
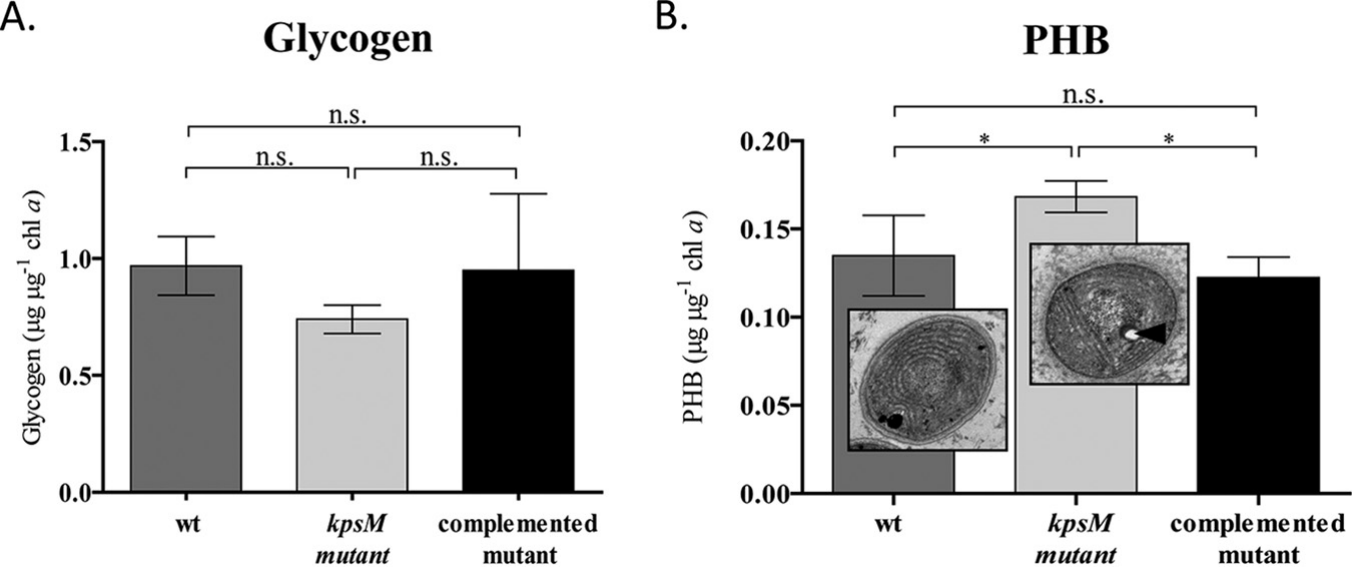
Quantification of glycogen and polyhydroxybutyrate (PHB) in *Synechocystis* sp. PCC 6803 wild-type (wt), *kpsM* mutant, and complemented mutant strains. (A) Glycogen quantification performed by the phenol-sulfuric acid assay and normalized by chlorophyll *a*. (B) PHB content determined by HPLC and normalized by chlorophyll *a*. The TEM micrographs show the ultrastructure of *Synechocystis* wild-type and *kpsM* mutant cells, with the arrowhead indicating the intracellular accumulation of PHB in the *kpsM* mutant. Cells were grown in BG11 medium at 30°C under a 12-h light (50 μE m^−2^ s^−1^)/12-h dark regimen, with orbital shaking at 150 rpm. Experiments were performed in triplicate, and statistical analysis is presented (n.s., not significant [*P* value of >0.05]; *, *P* value of ≤0.05).

To evaluate if the *kpsM* knockout mutant accumulates carbon intracellularly, the amount of carbon storage compounds was determined. Regarding glycogen, no significant differences were found between the mutant and the wild type/complemented mutant (Fig. 4A), representing about 4% of the dry-cell weight. In contrast, the *kpsM* mutant accumulates approximately 30% more polyhydroxybutyrate (PHB) than the wild type/complemented mutant (Fig. 4B). The PHB content represents about 1% of the dry-cell weight for the wild type. Moreover, cultivation under conditions that potentiate the accumulation of PHB (nitrate-free medium [BG11_0_]) (28) led to an increase in the PHB content in both the *kpsM* mutant and the wild type (Fig. S3).

10.1128/mSphere.00003-21.4 FIG S3 Quantification of the PHB content of *Synechocystis* sp. PCC 6803 wild-type (wt) and *kpsM* mutant strains by Nile red-based fluorescence spectroscopy. (A) Cells cultured in BG11 medium at 30°C under a 12-h light (50 μE m^−2^ s^−1^)/12-h dark regimen, with orbital shaking at 150 rpm. (B) Cells cultured in BG11_0_ medium at 30°C under a 12-h light (50 μE m^−2^ s^−1^)/12-h dark regimen, with orbital shaking at 150 rpm for 5 days. Experiments were performed in triplicate, and statistical analysis is presented (n.s., not significant [*P* value of >0.05]; *, *P* value of <0.05). Download FIG S3, TIF file, 0.04 MB. Copyright © 2021 Santos et al. 2021 Santos et al. This content is distributed under the terms of the Creative Commons Attribution 4.0 International license.

### The extracellular medium of the *kpsM* mutant contains fewer carotenoids.

**FIG 5:**
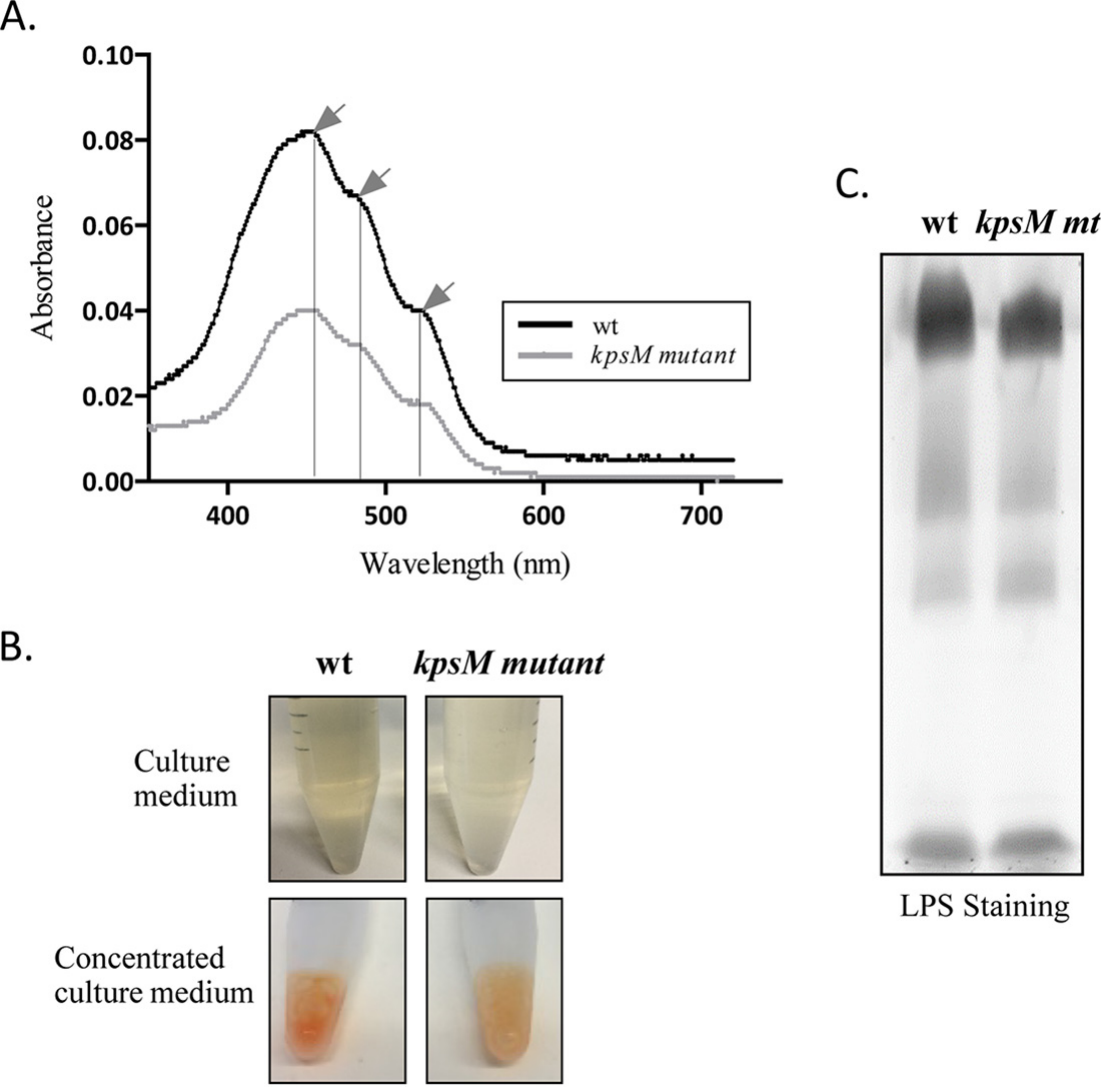
Analysis of the extracellular medium from *Synechocystis* sp. PCC 6803 wild-type (wt) and *kpsM* mutant cultures. (A) Absorption spectra of concentrated medium, with arrows indicating the characteristic carotenoid peaks (at 460, 487, and 521 nm). (B) *Synechocystis* wild-type and *kpsM* mutant culture media exhibiting different orange color intensities due to the amounts of carotenoids. (C) Analysis of the lipopolysaccharides (LPS) in the extracellular culture medium of *Synechocystis* wild-type and *kpsM* mutant (*mt*) strains by SDS-polyacrylamide gel electrophoresis followed by staining with Pro-Q Emerald 300 lipopolysaccharide.

While performing the RPS quantification, a clearly visible difference between the colors of the cell-free media from the wild type and the *kpsM* mutant was observed (Fig. 5B). Therefore, the pigment contents in the extracellular media of both cultures were analyzed. The absorption spectra of the concentrated samples showed the characteristic peaks of carotenoids for both strains although with a significantly higher content for the wild type (Fig. 5A), with the intracellular content not varying considerably (Fig. S4). For the complemented mutant, the intracellular and extracellular carotenoid contents are slightly higher than those of the wild type (Fig. S4). As carotenoids are lipophilic molecules and, thus, embedded in lipid structures, the presence of lipopolysaccharides (LPS) was also investigated. However, no significant differences were observed in terms of the LPS profile (Fig. 5C).

10.1128/mSphere.00003-21.5 FIG S4 Analysis of the intracellular and extracellular medium carotenoid contents of *Synechocystis* sp. PCC 6803 wild-type (wt), *kpsM* mutant, and complemented mutant (comp mutant) strains. Shown are absorption spectra of cell-free extracts (intracellular content) (A) and concentrated medium (B). Black arrows indicate the characteristic carotenoid (Car) peaks (at 460, 487, and 521 nm), while the gray arrow indicates the absorption peak of chlorophyll *a* (Chl a). Download FIG S4, TIF file, 0.1 MB. Copyright © 2021 Santos et al. 2021 Santos et al. This content is distributed under the terms of the Creative Commons Attribution 4.0 International license.

### The *kpsM* mutant has altered protein secretion.

**FIG 6:**
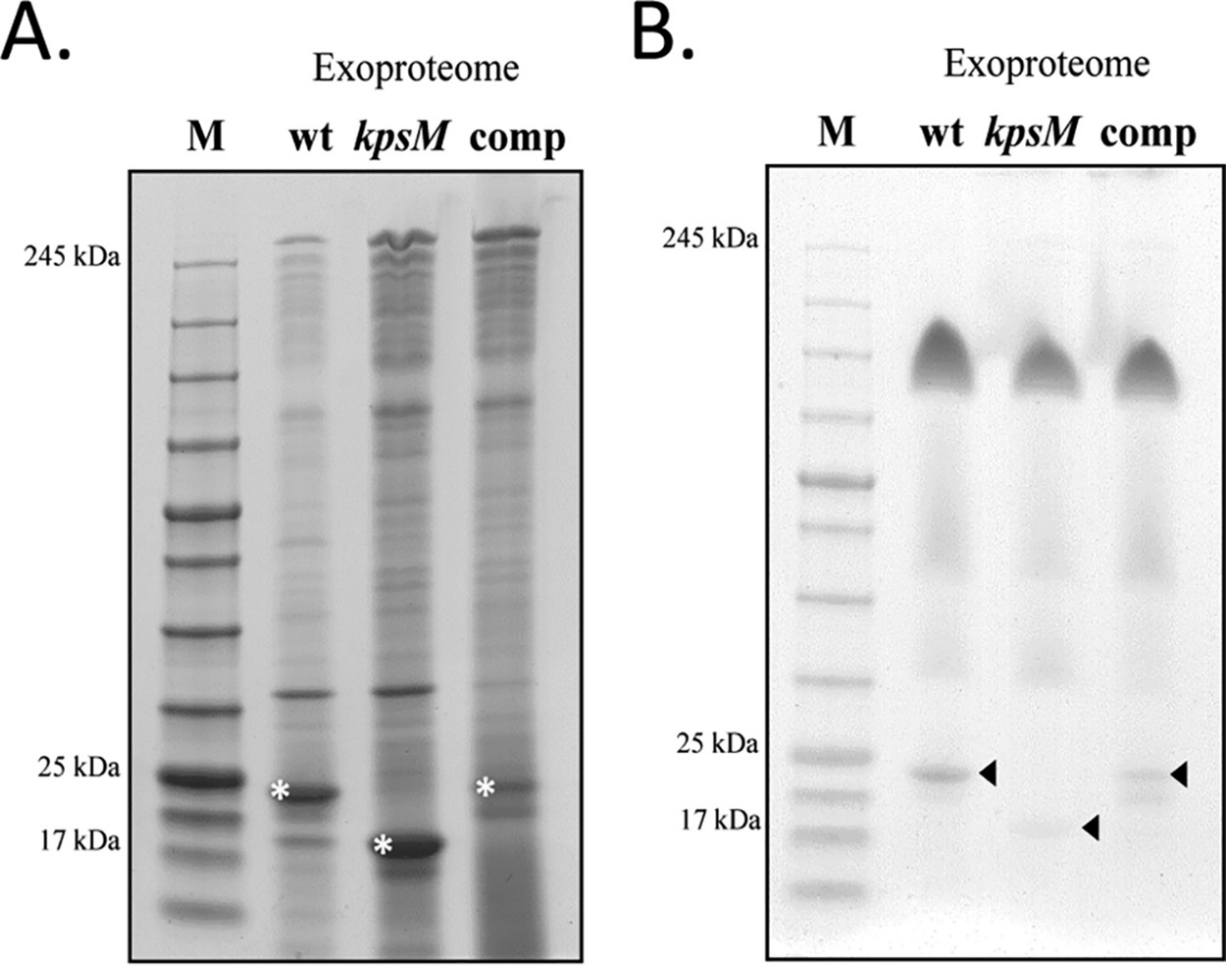
Exoproteomes of *Synechocystis* sp. PCC 6803 wild-type (wt), *kpsM* mutant, and complemented mutant strains. (A) Analysis of the proteins separated by SDS-PAGE and stained with Roti-Blue. Bands highlighted with an asterisk were observed across at least three biological replicates and excised for protein identification. (B) Analysis of the glycoproteins by SDS-PAGE followed by staining with a glycoprotein staining kit (Pierce). Arrowheads indicate glycosylation differences in the PilA component. Sample loading was normalized to each culture cell density (OD_730_), volume of cell-free medium concentrated, and concentration factor. M, NZYColour protein marker II (NZYTech).

Taking into consideration the differences observed due to the amount of carotenoids, further characterization of the extracellular media was undertaken. First, the exoproteomes of the wild type, the *kpsM* mutant, and the complemented strain were analyzed to unveil possible alterations in protein secretion. The *kpsM* mutant releases more proteins into the medium than the wild type (Fig. 6A). In addition, although the in-gel profiles of the exoproteomes of the three strains were overall similar, it was possible to observe a strong band with a molecular mass of approximately 22 kDa for the wild type and a similar but weaker band for the complemented mutant, while a band with a molecular mass of approximately 17 kDa was observed for the *kpsM* mutant (Fig. 6A). These proteins were identified by mass spectrometry and contained peptides corresponding to the pilus component PilA. We hypothesized that the molecular mass shift observed for the *kpsM* mutant could be due to alterations in posttranslational modifications of PilA, namely, glycosylation. To validate this, the exoproteome samples were stained with a glycoprotein staining kit (Pierce). The band identified in the wild type and the complemented mutant as PilA was glycosylated, while differential glycosylation occurred in the region of the PilA-corresponding band in the *kpsM* mutant (Fig. 6B). In addition, the intracellular and outer membrane proteome profiles of the wild type and the *kpsM* mutant were also analyzed. Overall, these profiles were similar between the two strains (Fig. S5), with no significant differences observed.

10.1128/mSphere.00003-21.6 FIG S5 Total protein and outer membrane extracts of *Synechocystis* sp. PCC 6803 wild-type (wt) and *kpsM* mutant strains. Roti-Blue-stained 4 to 15% SDS-polyacrylamide gels of total protein extracts (A) and outer membrane protein extracts (B) are shown. Sample loading was normalized to the amount of protein. Arrowheads highlight bands corresponding to the S-layer protein component Sll1951 (5). M, NZYColour protein marker II (NZYTech). Download FIG S5, TIF file, 0.1 MB. Copyright © 2021 Santos et al. 2021 Santos et al. This content is distributed under the terms of the Creative Commons Attribution 4.0 International license.

### Absence of KpsM has a pleiotropic effect on *Synechocystis* homeostasis.

**FIG 7:**
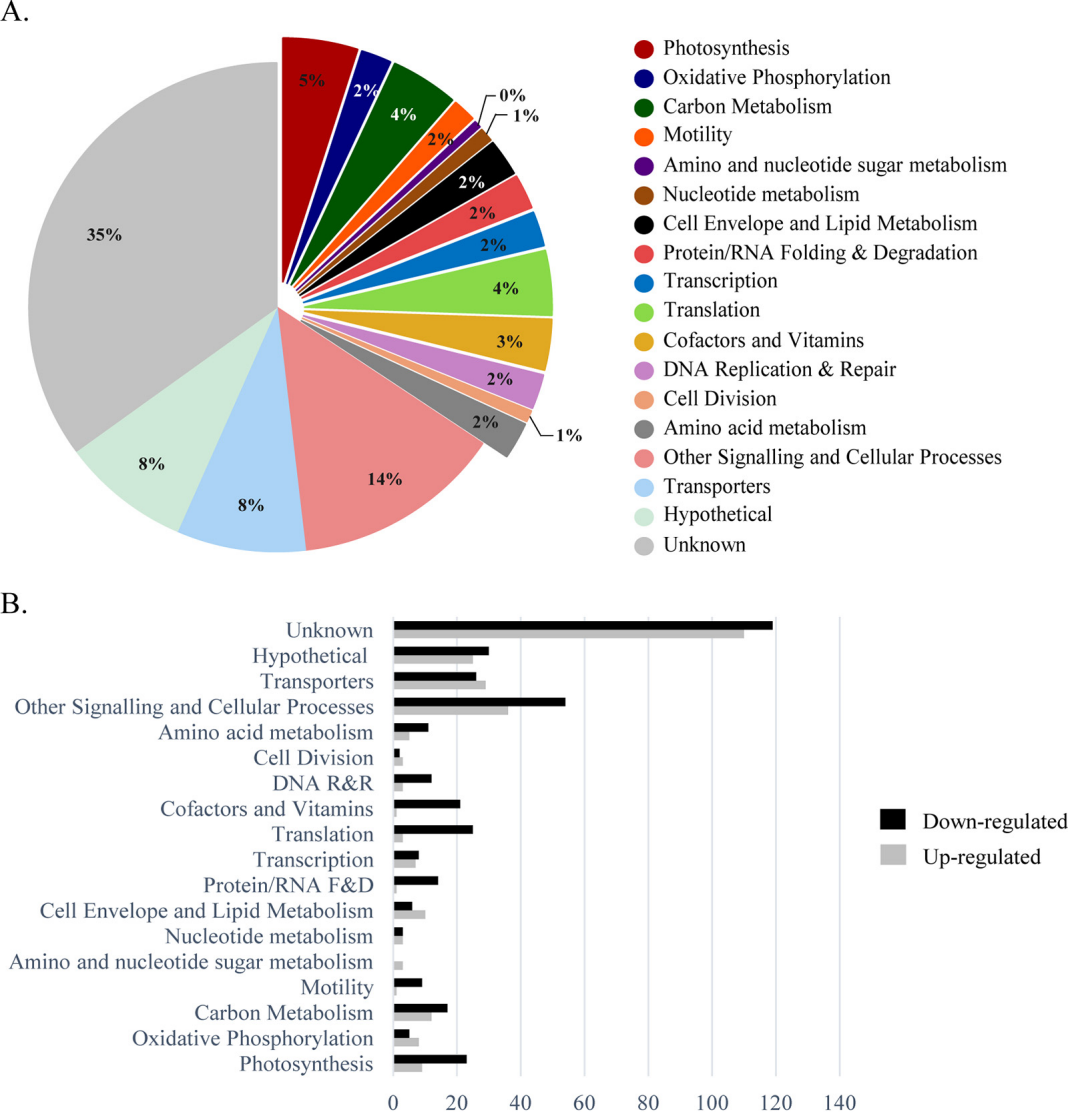
Functional groups of genes with transcript levels with significant fold changes in the *Synechocystis kpsM* mutant versus the wild type. (A) Distribution by functional category of the genes identified and quantified with significant fold changes in the RNA-seq analysis. (B) Number of genes up- or downregulated in the *kpsM* mutant compared to the wild type by functional category. See the annotated list of genes in Table S3 in the supplemental material. Functional categories were assigned based on information available at the CyanoBase and KEGG databases. R&R, replication and repair; F&D, folding and degradation.

**FIG 8:**
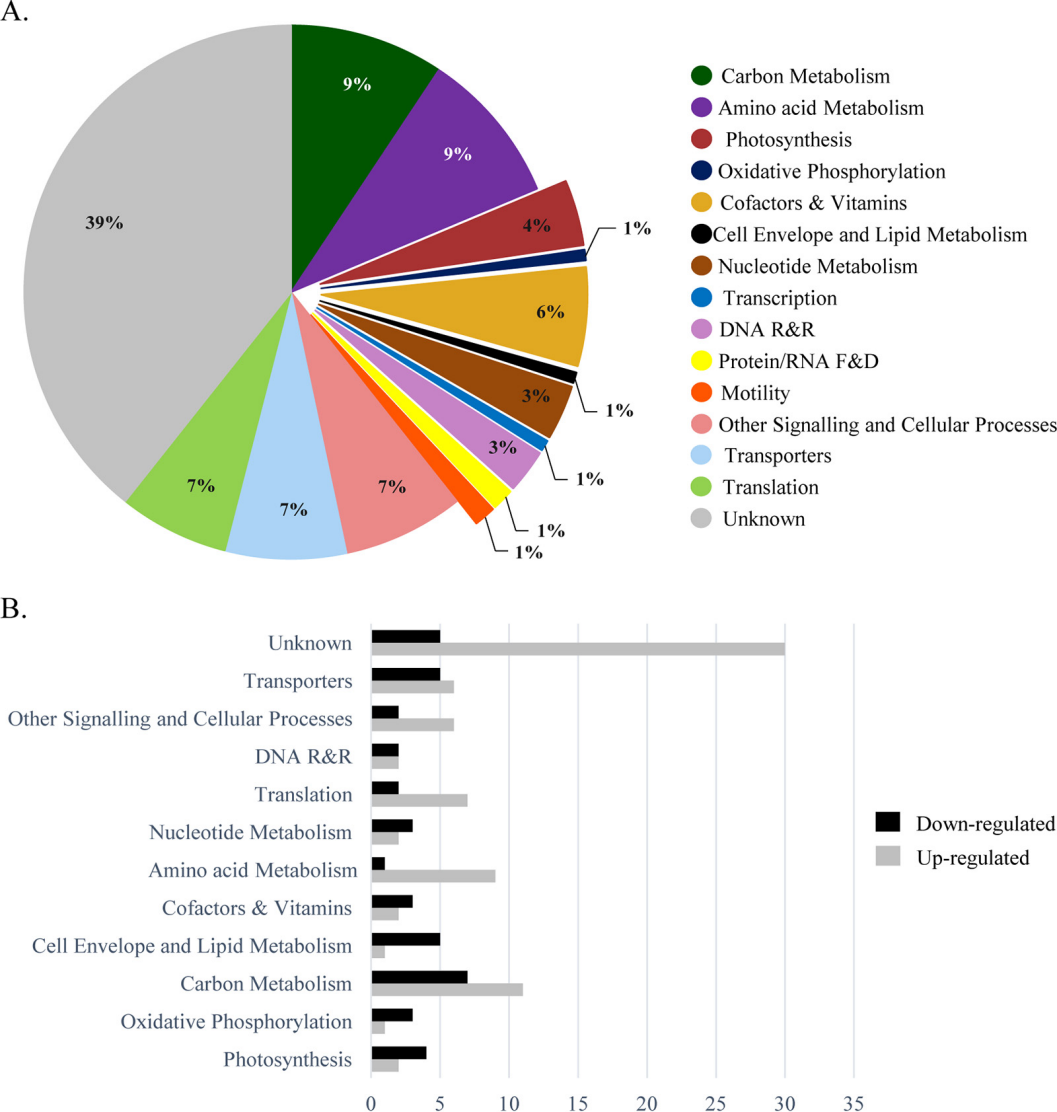
Functional groups of proteins with significant fold changes in the *Synechocystis kpsM* mutant versus the wild type. (A) Distribution by functional category of the proteins identified and quantified by iTRAQ analysis. (B) Number of proteins by functional category with significantly higher or lower abundances in the *kpsM* mutant than in the wild type. See the annotated list of proteins in Table S4 in the supplemental material. Functional categories were assigned based on information available at the CyanoBase and KEGG databases.

To obtain an overview of the metabolic changes associated with the absence of KpsM in *Synechocystis*, in particular in carbon-related metabolic pathways, two different high-throughput analyses were performed, namely, (i) whole-transcriptome analysis by RNA sequencing (RNA-seq) (Fig. 7) and (ii) isobaric tags for relative and absolute quantification (iTRAQ)-based quantitative proteomic analysis (Fig. 8). The genes and proteins referred to throughout this section are listed in Tables 1 and 2, respectively. The full lists of gene transcripts and proteins showing significant fold changes between the *kpsM* mutant and the wild type are available in Tables S1 and S2 in the supplemental material, respectively.

**TABLE 1:** Distribution by functional category of the genes presented in Results, quantified in the RNA-seq analysis with significant fold changes in mRNA transcript levels in the *Synechocystis kpsM* mutant versus the wild type

Functional category and ORF	Gene ID(s)	Description	Fold change (mutant/wt)
Photosynthesis			
*ssr2831*	*psaE*	Photosystem I reaction center subunit IV	1.8
*sll1194*	*psbU*	Photosystem II 12-kDa extrinsic protein	−1.6
*ssr0390*	*psaK1*	Photosystem I reaction center subunit X	1.7
*ssr3451*	*psbE*	Cytochrome *b*_559_ alpha subunit	−1.6
*sml0008*	*psaJ*	Photosystem I reaction center subunit IX	1.7
*sll0629*	*psaK*	Photosystem I subunit X	1.6
*sll0427*	*psbO*	Photosystem II manganese-stabilizing polypeptide	−1.5
*smr0004*	*psaI*	Photosystem I subunit VIII	1.6
	*psbZ*	Photosystem II	−1.5
*sll0819*	*psaF*	Photosystem I reaction center subunit III (PSI-F)	1.4
*sll1867*	*psbA3*	Photosystem II D1 protein	−2.1
*smr0008*	*psbJ*	Photosystem II PsbJ protein	−2.0
*ssl0563*	*psaC*	Photosystem I subunit VII	−1.3
*smr0005*	*psaM*	Photosystem I PsaM subunit	1.4

Oxidative phosphorylation			
*sll1324*	*atpF*	ATP synthase subunit b	2.2
*sll1323*	*atpG*	ATP synthase subunit b′	2.2
*sll0223*	*ndhB*	NAD(P)H dehydrogenase I subunit 2	1.5
*sll1322*	*atpI*	ATP synthase subunit a	1.7
*sll1325*	*atpD*	ATP synthase d subunit	1.8
*sll0522*	*ndhE*	NADH dehydrogenase subunit 4L	−1.7
*slr0851*	*ndh*	NADH dehydrogenase	1.4

Carbon metabolism			
*slr1945*	*pgm*	2,3-Bisphosphoglycerate-independent phosphoglycerate mutase	1.6
*slr1843*	*zwf*	Glucose-6-phosphate 1-dehydrogenase	1.4

Motility			
*slr1276*		Type IV pilus assembly protein PilO	−1.7
*slr1275*		Type IV pilus assembly protein PilN	−1.8
*sll1694*	*hofG*	General secretion pathway protein G	−1.5
*sll1533*	*pilT*	Twitching motility protein	−1.8
*sll1291*		Twitching motility two-component system response regulator PilG	2.0

Amino sugar and nucleotide sugar metabolism			
*slr0017*	*murA*	UDP-*N*-Acetylglucosamine 1-carboxyvinyltransferase	2.8
*slr1746*	*murI*	Glutamate racemase	3.0
*slr1423*	*murC*	UDP-*N*-Acetylmuramate-alanine ligase	2.2

Transcription			
*sll1689*	*sigE*; *rpoD*	Sigma factor E	1.8

Amino acid metabolism			
*slr0528*	*murE*	UDP-MurNAc-tripeptide synthetase	1.7

Transporters			
*sll0923*	*epsB*; *wzc*	Exopolysaccharide export protein	2.0
*sll1581*	*gumB*	Polysaccharide biosynthesis/export	1.8

Other signaling and cellular processes			
*slr0328*	*wzb*	Low-mol-wt protein-tyrosine phosphatase	−2.7

**TABLE 2:** Distribution by functional category of the proteins presented in Results, quantified by iTRAQ analysis with significant fold changes in the *Synechocystis kpsM* mutant versus the wild type

Functional category and protein name (alternative name[s])	UniProt accession no.	Description^*a*^	Fold change (mutant/wt)
Oxidative phosphorylation			
Sll1325 (AtpH; AtpD)	P27180	ATP synthase d subunit	1.5

Carbon metabolism			
Ssl2501 (PhaP)	P73545	Phasin (GA13)	3.1
Sll1070 (TktA)	P73282	Transketolase (EC 2.2.1.1)	1.4
Slr1945 (GpmI; Pgm)	P74507	iPGM (EC 5.4.2.12)	1.3
Slr0752 (Eno)	P77972	Enolase (EC 4.2.1.11) (2-phospho-d-glycerate hydrolyase) (2-phosphoglycerate dehydratase)	1.2

Cell envelope and lipid metabolism			
Sll1951	P73817	S-layer protein (HLP)	2.7

Cofactors and vitamin metabolism			
Slr1055 (ChlH)	P73020	Mg-chelatase subunit ChlH (anti-sigma factor E)	−1.3

Other signaling and cellular processes			
Slr1963	P74102	OCP	1.4
Slr0088 (CrtO)	Q55808	β-Carotene ketolase	−1.3
Sll0254	P73872	Carotenoid phi-ring synthase	−1.7

a iPGM, 2,3-bisphosphoglycerate-independent phosphoglycerate mutase; HLP, hemolysin-like protein; OCP, orange carotenoid binding protein.

10.1128/mSphere.00003-21.7 TABLE S1 Distribution by functional category of the genes quantified in the RNA-seq analysis with significant fold changes in mRNA transcript levels in the *Synechocystis kpsM* mutant versus the wild type. Download Table S1, DOCX file, 0.1 MB. Copyright © 2021 Santos et al. 2021 Santos et al. This content is distributed under the terms of the Creative Commons Attribution 4.0 International license.

10.1128/mSphere.00003-21.8 TABLE S2 Distribution by functional category of the proteins quantified in the iTRAQ analysis with significant fold changes in the *Synechocystis kpsM* mutant versus the wild type. Download Table S2, DOCX file, 0.03 MB. Copyright © 2021 Santos et al. 2021 Santos et al. This content is distributed under the terms of the Creative Commons Attribution 4.0 International license.

10.1128/mSphere.00003-21.9 TABLE S3 List of organisms and plasmids used/generated in this work. Download Table S3, DOCX file, 0.01 MB. Copyright © 2021 Santos et al. 2021 Santos et al. This content is distributed under the terms of the Creative Commons Attribution 4.0 International license.

10.1128/mSphere.00003-21.10 TABLE S4 Oligonucleotides used in this work. Download Table S4, DOCX file, 0.01 MB. Copyright © 2021 Santos et al. 2021 Santos et al. This content is distributed under the terms of the Creative Commons Attribution 4.0 International license.

Regarding RNA sequencing, approximately 700 genes (out of the 3,636 identified and quantified, representing a coverage of 85.12%) were significantly differentially expressed (*P* value of <0.05) in the *kpsM* mutant compared to the wild type. Of those, 406 were downregulated, while 297 were upregulated. The highest percentage (43%) belongs to the “unknown” and “hypothetical” categories, consistent with the fact that nearly half of the *Synechocystis* genome is still annotated as hypothetical. The other most represented functional groups include the broad-range category “other signaling and cellular processes” and “transporters” (Fig. 7A). Moreover, it is important to highlight the considerable changes observed in the levels of transcripts related to the mechanisms of energy production and conversion, including “photosynthesis,” “carbon metabolism,” and “oxidative phosphorylation.” In addition, “translation” and “photosynthesis” were two of the functional categories with the highest numbers of downregulated genes (Fig. 7B), indicating a strong effect of the absence of KpsM on the translational mechanisms and significant changes in the photosynthetic machinery of the mutant. In agreement, several *psb* genes (encoding components of photosystem II) were downregulated, whereas a number of those coding for photosystem I constituents, *psa*, were upregulated in the *kpsM* mutant. Notably, inactivation of *kpsM* also affects the transcript levels of genes putatively related to the Wzy-dependent pathway of EPS assembly and export, with upregulations of 1.8- and 2.0-fold of *wza* and *wzc*, respectively, and a downregulation of 2.7-fold of *wzb* (gene encoding a low-molecular-weight phosphatase). In addition, genes encoding proteins related to the cell surface/cell wall, namely, the *pil* components (*pilA*, *pilN*, *pilO*, and *pilT*) involved in pilus biogenesis and motility, were downregulated, while the *mur* genes, associated with peptidoglycan biosynthesis, were upregulated.

Regarding the main players in oxidative phosphorylation, the transcript levels of NADH dehydrogenase and ATPase subunits (f, g, i, and d) were significantly higher in the mutant (Table 1). Furthermore, *zwf* (*slr1843*), encoding the key player in the oxidative pentose phosphate pathway (OxPPP), glucose-6-phosphate dehydrogenase, was upregulated 1.4-fold in the mutant. In agreement, an upregulation of ∼2-fold of the transcript levels of the gene encoding sigma factor E was also observed.

The iTRAQ-based quantitative proteome analysis led to the identification and quantification of 1,675 proteins (coverage, 47.7%). Statistically significant fold changes (*P* value of <0.05) were found for 150 proteins. Of these, the levels of 79 were significantly higher whereas those of 71 were significantly lower in the mutant than in the wild type. The distribution of these proteins by functional category is similar to that observed for the gene transcripts, with “unknown” being the most represented, comprising 38 and 41% of the proteins with higher and lower abundances, respectively. Other strongly represented functional categories included “carbon metabolism,” “nucleotide metabolism,” “transporters,” “other signaling and cellular processes,” and “translation” (Fig. 8A). The distribution by functional category of the proteins displaying higher or lower abundances in the mutant is shown in Fig. 8B. Notably, phasin (PhaP), which accumulates together with PHB granules, was found to be more abundant in the mutant (3.1-fold), in agreement with the mutant accumulating more PHB than the wild type. The absence of KpsM also led to an increase of 2.7-fold in the abundance of the S-layer protein Sll1951 in the mutant. In addition, the d subunit of the ATP synthase (Sll1325) was 1.5-fold more abundant in the mutant than in the wild type, in agreement with the results obtained by RNA sequencing. Furthermore, two proteins involved in carotenoid biosynthesis were found at lower abundances in the mutant than in the wild type. These proteins were the β-carotene ketolase CrtO (Slr0088) (1.3-fold less abundant) and the carotenoid phi-ring synthase Sll0254 (1.7-fold less abundant). Additionally, the orange carotenoid binding protein (OCP) was found at a higher abundance in the mutant than in the wild type (1.4-fold).

Regarding carbon metabolism, we observed increases in the levels of the enzymes involved in the sugar catabolic pathways, namely, the transketolase TktA (Sll1070), the phosphoglycerate mutase Pgm (Slr1945), and the enolase Eno (Slr0752) (Table 2). Relevantly, a smaller amount of the anti-sigma factor E enzyme ChlH (Slr1055) (29) was observed in the mutant than in the wild type, whereas the levels of SigE did not change significantly.

Previous studies found that the correlation between transcriptomes and proteomes across large data sets was somewhat modest (30). Nevertheless, it was also described that the cellular processes/functional categories identified by the transcriptomic and proteomic analyses can be very similar (30, 31), which is in agreement with our data sets.

**FIG 9:**
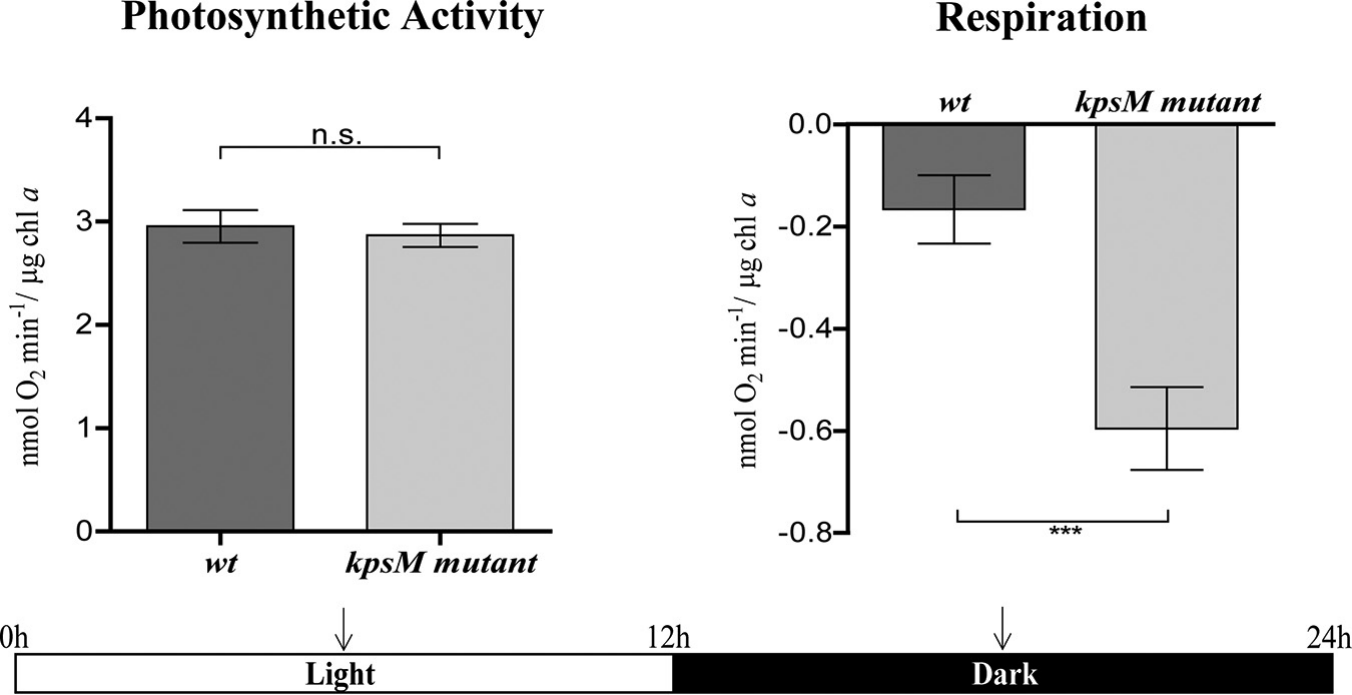
Oxygen evolution and consumption rates by *Synechocystis* sp. PCC 6803 wild-type (wt) and *kpsM* mutant strains. Photosynthetic activity was measured in the middle of the light period, and respiration was measured in the middle of the dark period (arrows) of the 12-h light/12-h dark growth regimen as described in Materials and Methods (*, *P* value of ≤0.05; **, *P* value of ≤0.01).

Due to the observed differences in the transcriptomic and proteomic data regarding photosynthetic mechanisms and cell wall/surface components between the *kpsM* mutant and the wild type, the O_2_ evolution/consumption rates and the cell envelope ultrastructures of the two strains were evaluated. The mutant showed a similar O_2_ evolution rate during the light period and a higher O_2_ consumption rate during the dark period (Fig. 9).

**FIG 10:**
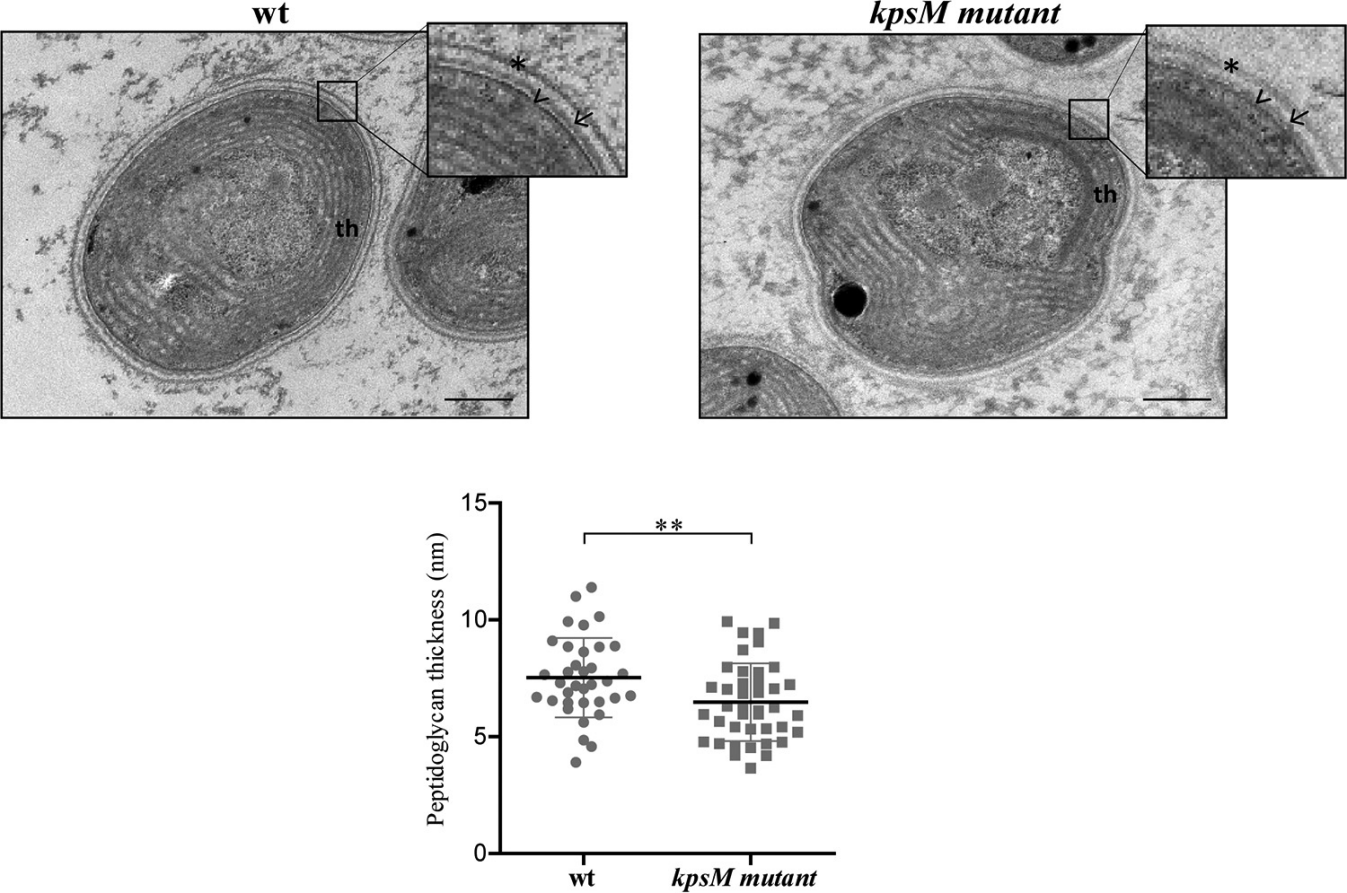
Ultrastructure of *Synechocystis* sp. PCC 6803 wild-type (wt) and *kpsM* mutant cells. The inset panels show details of the cell walls. Asterisk, S layer; arrow, outer membrane; arrowhead, peptidoglycan; th, thylakoids. Bars, 100 nm. The measurements of peptidoglycan thickness are presented below the micrographs (**, *P* value of ≤0.01).

Furthermore, no noticeable differences were observed for the cell envelope, except for a small but significant difference in the peptidoglycan thickness (mutant, 6.48 ± 1.7 nm; wild type, 7.53 ± 1.6 nm) (Fig. 10).

## DISCUSSION

The thorough characterization of the *Synechocystis slr0977* (*kpsM*) knockout mutant performed here shows that the absence of the putative transport permease has a pleiotropic effect on a variety of cellular processes. Although a *Synechocystis slr0977* mutant was generated previously by Fisher et al. (3), we chose to generate a mutant using the Kazusa substrain (32) to allow direct comparison with several EPS-related mutants previously generated in our laboratory (26). In agreement with the results obtained by Fisher et al. (3), no significant growth differences were observed between the *kpsM* mutant and the wild type, and the presence of a flocculent phenotype was also noticed, suggesting a light-sensitive phenotype. In addition to the previously reported differences in the EPS monosaccharidic composition (3), our results clearly show that the absence of KpsM leads to an overall reduction of EPS production, with the mutant having a significant reduction of RPS (50% after 21 days of culture) and a less pronounced decrease of CPS (about 20%). Complementation of the mutant with native *kpsM* (*kpsM* mutant::p351slr0977) restored EPS production to levels that even surpassed the ones of the wild type (see Fig. S2 in the supplemental material), with this most likely being due to the use of a medium-strength promoter, psbA2* (33) instead of the native one. Interestingly, another *Synechocystis* mutant generated in our laboratory in another putative *kpsM* homologue (*slr2107*) shows no differences regarding EPS production compared to the wild type (26), suggesting that, at least under the conditions tested, Slr2107 does not play a major role in EPS production. Interestingly, the EPS produced by the triple mutant (*slr0977* [*kpsM*] *sll0574* [*kpsM*] *sll0575* [*kpsT*]) generated by Fisher et al. has a composition similar to that of the wild type, leading the authors to hypothesize the use of different transport components or an alternative route (3). Previous works in *Synechocystis* demonstrated that the putative Wzy-dependent components Wza, Wzb, and Wzc are also involved in EPS production (25, 26). The deletion of *wza* results in a substantial decrease in CPS, with no effect on RPS production, while the deletion of *wzb* results in a decrease in RPS only, and the deletion of *wzc* decreases the amounts of both RPS and CPS produced. However, different *Synechocystis* substrains and culture conditions were used in the two studies, not allowing a direct comparison of the results. Since the *Synechocystis* substrain and the experimental design used here were the same as the ones used previously by Pereira et al. (26), we can infer that deletion of *kpsM* results in one of the most significant reductions of the amount of RPS reported to date.

In other bacteria, in the Wzy-dependent pathway, Wzc undergoes a phosphorylation/dephosphorylation cycle that affects its oligomerization state and is dependent on the phosphatase activity of Wzb (34). Recently, Pereira et al. (26) also showed that in *Synechocystis*, and at least *in vitro*, Wzc is a substrate of Wzb, suggesting a possible regulatory role for the low-molecular-weight phosphatase Wzb. In agreement, the transcriptomic data obtained here show the upregulation of *wza* and *wzc* and the downregulation of *wzb* in the *Synechocystis kpsM* mutant, although no significant differences were observed regarding the abundances of the corresponding proteins. These results reinforce the indirect role of *wzb* in cyanobacteria as in other bacteria. The transcriptional response of these putative Wzy-dependent components raises the hypothesis that the mutant attempts to balance the absence of *kpsM* either by using a distinct export route or even by using other components, as the two canonical bacterial pathways might not function as separate entities in *Synechocystis*/cyanobacteria.

Our results also showed that in the *kpsM* mutant, the decrease of EPS goes together with the intracellular accumulation of carbon in the form of the storage compound polyhydroxybutyrate (PHB). Supporting this result, the phasin PhaP, a protein that is known to accumulate on the surface of PHB granules (35), was found in a higher abundance in the mutant than in the wild type. Previous work suggested that the accumulation of PHB is a direct result of glycogen turnover during nitrogen depletion in *Synechocystis* (36–38). It is important to highlight that the culture conditions used throughout this study are always those of nitrogen sufficiency and that the *kpsM* mutant does not show a statistically significant decrease in glycogen accumulation compared to the wild type; therefore, it is not possible to make the same inference from our work. Interestingly, the level of EPS produced by the *Synechocystis* wild-type strain (∼6 μg μg^−1^ chlorophyll *a*) is approximately 6-fold higher than that of the most commonly abundant intracellular carbon storage compound, glycogen (∼1 μg μg^−1^ chlorophyll *a*) (Fig. 2B and C and Fig. 3A). This oftentimes-overlooked fact suggests that EPS can act as an effective carbon sink in cyanobacteria. Lau et al. (39) previously reported that the main driving force for the synthesis of PHB in *Synechocystis* is the total flux of carbon. In agreement, the *kpsM* mutant shows differences in sugar metabolism/catabolism pathways compared to the wild type, including the upregulation of *sigE* and the lower abundance of the anti-sigma factor E enzyme ChlH. SigE was previously described as a positive transcriptional regulator of sugar catabolic pathways in *Synechocystis* (36, 40–42), with its activity being inhibited by ChlH (29). The results of RNA sequencing and iTRAQ analyses also showed that players involved in sugar catabolic pathways, including glycolysis and the oxidative pentose phosphate pathway (OxPPP), are present in higher abundances in the mutant (Tables 1 and 2). Among these is the upregulated phosphoglycerate mutase Pgm (Slr1945), operating at the beginning of lower glycolysis. Recently, this protein was proposed to play a key role in the regulation of cyanobacterial carbon storage metabolism (43). It was suggested that the higher carbon flux through lower glycolysis results in higher pyruvate levels, thereby increasing the amount of PHB (43). Similarly, Lau et al. (39) had suggested that an increment of sugar catabolism pathways likely results in a higher abundance of cellular metabolites that can be used as precursors for the synthesis of PHB. Moreover, other players involved in sugar catabolism uncovered by our transcriptomic and proteomic analyses comprise the OxPPP key component *zwf*, the transketolase TktA, and a second protein involved in lower glycolysis, the enolase Eno. These results are in agreement with those described previously by Tokumaru et al. (42), who reported the upregulation of players involved in sugar catabolism in a *Synechocystis* SigE overexpression mutant.

The high numbers of changes observed in the transcriptome and proteome of the *kpsM* mutant related to central energy and carbon metabolism seem to be correlated with a higher respiratory (O_2_ consumption) rate. However, the growth rates are similar between the mutant and the wild type, suggesting that the differences observed do not affect growth under standard laboratory conditions and thus suggesting that these physiological adjustments do not impact biomass formation.

The smaller amount of carotenoids present in the extracellular medium of the *kpsM* mutant and the lower levels of the β-carotene ketolase CrtO (Slr0088) and the carotenoid phi-ring synthase Sll0254 point toward an impairment in the carotenoid biosynthetic pathways. These results, together with the smaller amount of RPS, may explain the observed light-dependent clumping phenotype observed for the mutant. This mechanism provides self-shading for the cells, which may attenuate the absence of protection conferred by the carotenoids and extracellular polysaccharides (1, 44, 45). Furthermore, Slr1963 (encoding the orange carotenoid binding protein [OCP]) is more abundant in the mutant, suggesting a photoprotective mechanism in the *kpsM* mutant since it was previously described that OCP is an essential player in the stress response to high-light conditions by interacting with the phycobilisome by increasing energy dissipation in the form of heat, thereby decreasing the amount of energy arriving at the reaction centers and preventing an excess of reactive oxygen species (ROS) (46–48). Moreover, in other bacteria, biosynthesis and accumulation of PHB can be used as a mechanism to maintain the redox equilibrium in the cell by allowing the elimination of excess acetyl-CoA and reducing equivalents (49). Thus, the accumulation of PHB in the *kpsM* mutant could be another strategy to relieve oxidative stress, in parallel with the increase in the level of OCP and the clumping observed at low cell densities.

In line with results obtained by Fisher et al. (3), no differences were observed between the LPS profiles of the mutant and the wild type, supporting that disruption of *kpsM* does not alter the structure of the O-antigen (OAg) and thus solidifying the role of KpsM as an extracellular polysaccharide transporter. On the other hand, the higher accumulation of proteins in the extracellular medium of the mutant indicates that in the absence of KpsM, the protein secretion capacity is affected. Moreover, the glycosylation pattern of the pilus component PilA present in the exoproteome is altered. Gonçalves et al. (50) reported differential pilin glycosylation in the PilA1 component of knockout mutants lacking proteins associated with the TolC-dependent secretion mechanisms. Gonçalves et al. (50) suggest that the deleted proteins could be involved in the processing and/or secretion of different extracellular proteins, thus affecting PilA1. In the case of the *kpsM* mutant, differences in the PilA glycosylation profile may be related to the role of KpsM in polysaccharide transport. Relevantly, differences in the transcripts levels of *pil* components and the *mur* genes and the higher abundance of the S-layer protein Sll1951 did not result in noticeable alterations of the cell envelope of the mutant compared to the wild type, except for a minor difference in peptidoglycan thickness. In agreement, previous work reported that a high rate of turnover of peptidoglycan components occurred when cells were light sensitive and, thus, more susceptible to photodamage (51, 52).

Overall, our transcriptomic and proteomic data indicate alterations in the mechanisms of energy production and conversion in the *kpsM* mutant compared to the wild type. Both approaches resulted in the identification of altered levels of transcripts and proteins belonging to the same functional categories, highlighting a number of key metabolic processes affected by the disruption of *kpsM*, namely, photosynthesis, oxidative phosphorylation, and carbon metabolism. In conclusion, we provide evidence of (i) the involvement of *Synechocystis* KpsM (Slr0977) in EPS export; (ii) the broad transcriptomic, proteomic, and, ultimately, physiological adaptation of *Synechocystis* cells to the absence of KpsM; and (iii) how a mutant impaired in the export of polysaccharides can redirect carbon flux toward the production of other carbon-based compounds, in particular PHB. Furthermore, in addition to the biological roles already described for cyanobacterial extracellular polysaccharides, the present work emphasizes the importance of cyanobacterial EPS as a carbon sink and shows how cells metabolically adapt when their secretion is impaired. Due to its fitness and accumulation of PHB, the *kpsM* mutant can also be used as a platform/chassis for the production of carbon-based compounds or other compounds of interest.

## MATERIALS AND METHODS

### Bacterial strains and culture conditions.

The cyanobacterium *Synechocystis* sp. PCC 6803 substrain Kazusa (Pasteur Culture Collection) used in this work is nonmotile and glucose tolerant (32, 53). *Synechocystis* wild-type and mutant strains (see Table S3 in the supplemental material) (58) were cultured in BG11 medium (54) at 30°C under a 12-h light (50 μmol photons m^−1^ s^−2^)/12-h dark regime with orbital agitation (150 rpm). For solid medium, BG11 medium was supplemented with 1.5% Noble agar (Difco), 0.3% sodium thiosulfate, and 10 mM TES [*N*-tris(hydroxymethyl)methyl-2-aminoethanesulfonic acid]-potassium hydroxide (KOH) buffer (pH 8.2). For the selection and maintenance of mutants, BG11 medium was supplemented with kanamycin (Km) (up to 700 μg ml^−1^), streptomycin (Sm) (up to 25 μg ml^−1^), and/or chloramphenicol (Cm) (up to 25 μg ml^−1^). The E. coli strains used were cultured at 37°C in LB medium supplemented with ampicillin (100 μg ml^−1^), Km (25 μg ml^−1^), and/or Cm (25 μg ml^−1^).

### Cyanobacterial DNA extraction and recovery.

Cyanobacterial genomic DNA was extracted using the Maxwell 16 system (Promega). For use in Southern blot analysis, the phenol-chloroform method described previously (55) was preferred. Agarose gel electrophoresis was performed according to standard protocols (56), and the DNA fragments were isolated from gels, enzymatic assay mixtures, or PCR mixtures using the NZYGelpure purification kit (NZYTech).

### Plasmid construction for *Synechocystis* transformation.

The *Synechocystis* chromosomal regions flanking *kpsM* (*slr0977*) were amplified by PCR using specific oligonucleotides (Table S4). An overlapping region containing an XmaI restriction site was included in primers 5I and 3I for cloning purposes. For each gene, the purified PCR fragments were fused by “overlap PCR.” The resulting products were purified and cloned into the vector pGEM-T Easy (Promega), creating plasmid pGDslr0977. A selection cassette containing the *nptII* gene (conferring resistance to neomycin and kanamycin) was amplified from pKm.1 using the primer pair Km.KmScFwd/KmRev (57) (Table S4) and digested with XmaI (Thermo Scientific). Subsequently, the purified selection cassette was cloned into the XmaI restriction site of the plasmids using the T4 DNA ligase (Thermo Scientific) to form pGDslr0977.Km.

The cassette containing the *aadA* gene (conferring resistance to streptomycin and spectinomycin) was obtained by digesting the plasmid pSEVA481 (57) with PshAI and SwaI, and the cassette was cloned in the XmaI/SmaI site of pGDslr0977 to form plasmid pGDslr0977.Sm.

For mutant complementation, the shuttle vector pSEVA351 (57) was used. A fragment covering the whole *kpsM* gene was amplified using primer pair slr0977Fwd_comp/sll0977Rev_comp (Table S2), purified from the gel, and digested with XbaI and PstI. The P_psbA2*_ promoter (33) and the synthetic ribosome binding site (RBS) BBa_B0030 were purified from the gel after digestion of plasmid pSBA2P_psbA2*_::B0030 with EcoRI and SpeI. The purified products were simultaneously cloned into pSEVA351 previously digested with EcoRI and PstI, creating plasmid pS351P_psbA2*_::B0030.*slr0977*. All constructs were verified by sequencing (StabVida) before transformation into *Synechocystis*.

### Generation of the *Synechocystis* sp. PCC 6803 mutants.

*Synechocystis* was transformed with integrative plasmids using a procedure described previously (59). Briefly, *Synechocystis* cultures were grown until the optical density at 730 nm (OD_730_) reached ∼0.5, and cells were harvested by centrifugation and suspended in a 1/10 volume of BG11 medium. Five hundred microliters of cells was incubated with 10 μg ml^−1^ plasmid DNA for 5 h before spreading them onto Immobilon-NC membranes (0.45-μm pore size; Millipore) resting on solid BG11 plates; plates were kept at 30°C under continuous light for 24 h. Membranes were transferred to selective plates containing 10 μg ml^−1^ of kanamycin. Transformants were observed after 1 to 2 weeks. For complete segregation, colonies were grown with increasing antibiotic concentrations. Nonintegrative plasmids were transferred to *Synechocystis* by electroporation, as described previously (33). Briefly, cells were washed with 1 mM HEPES buffer (pH 7.5). Afterwards, cells were resuspended in 1 ml HEPES, and 60 μl was mixed with 1 μg of DNA and electroporated with a Bio-Rad Gene Pulser instrument at a capacitance of 25 μF. The resistor used was 400 Ω for a time constant of 9 ms with an electric field of 12 kV cm^−1^. Immediately after the electric pulse, the cells were suspended in 1 ml BG11 medium and spread onto the Immobilon-NC membranes as described above. After 24 h, the membranes were transferred to selective plates containing 2.5 μg ml^−1^ of chloramphenicol before being grown with increasing antibiotic concentrations.

### Southern blotting.

Southern blot analyses were performed using genomic DNAs of the wild type and *kpsM* mutant clones digested with SpeI. DNA fragments were separated by electrophoresis on a 1% agarose gel and blotted onto an Amersham Hybond-N membrane (GE Healthcare). Probes were amplified by PCR and labeled using the primers indicated in Table S2 with the digoxigenin (DIG) DNA labeling kit (Roche Diagnostics GmbH) according to the manufacturer’s instructions. Hybridization was done overnight at 56°C, and digoxigenin-labeled probes were detected by chemiluminescence using CPD-star (Roche Diagnostics GmbH) in a Chemi Doc XRS^+^ imager (Bio-Rad).

### Growth assessment.

Growth measurements were performed by monitoring the OD at 730 nm (60) using a Shimadzu UVmini-1240 instrument (Shimadzu Corporation) and determining the chlorophyll *a* content as described previously (61). All experiments were performed with three technical and three biological triplicates. Data were statistically analyzed as described below.

### Determination of total carbohydrate content, RPS, and CPS.

Total carbohydrate and RPS contents were determined as described previously (62). For CPS quantification, the procedure was performed as described previously (26). Briefly, 5 ml of dialyzed cultures was centrifuged at 3,857 × *g* for 15 min at room temperature, suspended in water, and boiled for 15 min at 100°C to detach the CPS from the cells’ surface. After new centrifugation as described above, CPS were quantified from the supernatants using the phenol-sulfuric acid method (63). Total carbohydrate, RPS, and CPS were expressed as milligrams per liter of culture or normalized by the chlorophyll *a* content. All experiments were performed with three technical and three biological triplicates. Data were statistically analyzed as described below.

### Glycogen quantification.

Cells were collected at an OD_730_ of 1.5 and washed twice with BG11 medium. Glycogen was extracted as described previously (64, 65). Briefly, the pellet was suspended in ∼100 μl of double-distilled water (ddH_2_O), and 400 μl of 30% KOH was added. The mixture was incubated at 100°C for 90 min and then quickly cooled on ice. Six hundred microliters of ice-cold absolute ethanol was added, and the mixture was incubated on ice for 2 h and centrifuged for 5 min at maximum speed at 4°C. The supernatant was discarded, and the isolated glycogen isolated in the pellet was stored. Pellets were washed twice with 500 μl of ice-cold ethanol and dried at 60°C. Glycogen quantification was performed using the phenol-sulfuric acid assay (63).

### PHB quantification.

The PHB content was determined as described previously (37). Roughly 100 ml of cells was harvested and centrifuged at 4,000 × *g* for 10 min at 25°C. The resulting pellet was dried overnight at 80°C. About 30 mg of dried cells was boiled with 1 ml of H_2_SO_4_ at 100°C for 1 h to convert PHB to crotonic acid. After cooling, 100 μl was diluted with 900 μl of 0.014 M H_2_SO_4_, and the samples were centrifuged to remove cell debris for 10 min at 10,000 × *g*. Five hundred microliters of the supernatant was transferred to 500 μl of 0.014 M H_2_SO_4_. After an additional centrifugation step (the same conditions as the ones described above), the supernatant was used for high-performance liquid chromatography (HPLC) analysis. Commercially available crotonic acid was used as a standard with a conversion ratio of 0.893 (37). For the HPLC analysis, an ACE-C_18_ column (150- by 4.6-mm internal diameter [ID] with a particle size of 5 μm) (Advanced Chromatography Technologies Ltd.) was used. The HPLC system was equipped with a Shimadzu LC-20AD pump, a Shimadzu DGV-20A5 degasser, a Rheodyne 7725i injector fitted with a 100-μl loop, and an SPD-M20A diode array detection (DAD) detector. Data acquisition was performed using Shimadzu LCMS Lab Solutions software, version 3.50 SP2. The mobile phase composition was 20 mM phosphate buffer (NaH_2_PO_4_) (pH 2.5) and acetonitrile (95:5, vol/vol). All HPLC-grade solvents were purchased from Merck Life Science SLU. The flow rate was 0.85 ml min^−1^, and the UV detection wavelength was 210 nm. Analyses were performed at 30°C in an isocratic mode. Crotonic acid was purchased from Merck Life Science SLU, and serial dilutions were prepared in 0.014 M H_2_SO_4_ (0.1, 0.5, 1, 5, 10, 50, and 100 μM) to obtain the standard curve (retention time = 11.4 min; *y* = 123,225*x* + 152,755; *R*^2^ = 0.999). All samples were injected in duplicate.

### Outer membrane isolation and lipopolysaccharide staining.

Outer membranes were isolated as described previously by Simkovsky et al. (66). The pellet was suspended in 100 μl of 10 mM Tris-HCl (pH 8.0). Protease-digested samples were separated by electrophoresis on 12% SDS-PAGE gels (Bio-Rad), and lipopolysaccharide (LPS) was stained using a Pro-Q Emerald 300 lipopolysaccharide gel stain kit (Molecular Probes), according to the manufacturer’s instructions.

### Analysis of extracellular medium.

The medium from the *Synechocystis* wild-type and *kpsM* mutant cultures was isolated according to methods described previously by Oliveira et al. (67). Briefly, 100 ml of cultures was collected at an OD_730_ of ∼1.5 by centrifugation (4,000 × *g*). The supernatant was filtered through a 0.2-μm-pore-size filter and further concentrated by centrifugation with Amicon Ultra-15 centrifugal filter units (Merck Millipore) with a nominal molecular weight limit of 3 kDa. Concentrated exoproteome samples were then saved at −20°C until further analysis. Analysis of the exoproteomes was performed using concentrated medium samples. Exoproteome samples were separated by electrophoresis on 4-to-15% gradient SDS-polyacrylamide gels (Bio-Rad) and visualized with either Roti-Blue (Roth) or a glycoprotein staining kit (Pierce). Samples were normalized to the culture cell density (OD_730_), volume of cell-free culture medium concentrated, and concentration factor. Stained bands or gel regions observed consistently across at least three biological replicates were further excised and processed for mass spectrometry analysis as described previously (68, 69). Samples were reduced, alkylated, and further trypsin digested for obtaining the mass spectra by using a liquid chromatography-mass spectrometry (LC-MS) Orbitrap instrument. Protein identification was performed using the UniProt protein sequence database for the taxonomic selection *Synechocystis* (2017_01 release). Quantification of protein abundance was performed using the LFQ (label-free quantification) approach. For pigment analysis, absorption spectra in the visible light range (between 350 and 750 nm) were collected from concentrated exoproteome samples diluted 1:100 on a Shimadzu UV-2401 PC spectrophotometer (Shimadzu Corporation). For LPS detection, concentrated medium samples were separated by gel electrophoresis on 4 to 15% SDS-polyacrylamide gels (Bio-Rad), which were stained as mentioned above.

### Total protein and outer membrane protein profile analyses and iTRAQ experiments.

For total protein isolation, cells were harvested by centrifugation at 3,857 × *g* for 10 min at room temperature and washed in phosphate buffer (50 mM K_2_HPO_4_, 50 mM KH_2_PO_4_ [pH 6.9]). Cells were suspended in protein extraction buffer (50 mM Tris-HCl, 1 mM EDTA, 0.5% Triton X-100, 10% glycerol, 2 mM dithiothreitol [DTT], and 1 tablet of cOmplete EDTA-free protease inhibitor cocktail [Roche] per 10 ml of buffer), and proteins were extracted by mechanical cell disruption using a FastPrepR-24 cell disruptor, with an output of 6.5 m/s and 5 cycles of 30 s (MP Biomedicals), using glass beads (425 to 600 μm; Sigma-Aldrich), followed by centrifugation at 16,100 × *g* for 15 min at 4°C. Outer membranes were isolated as described above (see “Outer membrane isolation and lipopolysaccharide staining”). Protein preparations were stored at −80°C until further use. The protein concentration was determined using the bicinchoninic acid (BCA) protein assay kit (Pierce Biotechnology) and the iMark microplate absorbance reader (Bio-Rad) according to the manufacturers’ instructions. Samples were separated by electrophoresis as described above (see “Analysis of extracellular medium”). The proteomes of *Synechocystis* wild-type and *kpsM* mutant strains were analyzed by 8-plex isobaric tags for relative and absolute quantification (iTRAQ), using three biological replicates. A detailed description of the procedure can be found in Text S1 in the supplemental material. Descriptions of the proteins identified and their distributions into functional categories were based on data from the CyanoBase (http://genome.microbedb.jp/cyanobase) (70), UniProt (http://www.uniprot.org/), and KEGG (Kyoto Encyclopedia of Genes and Genomes) (http://www.genome.jp/kegg/) databases and complemented with the information available in the literature.

10.1128/mSphere.00003-21.1 TEXT S1 Experimental procedures. Detailed descriptions of the RNA sequencing and iTRAQ experiments are provided. Download Text S1, DOCX file, 0.03 MB. Copyright © 2021 Santos et al. 2021 Santos et al. This content is distributed under the terms of the Creative Commons Attribution 4.0 International license.

### RNA extraction and RNA sequencing.

For RNA extraction, the TRIzol reagent (Ambion) was used in combination with the PureLink RNA minikit (Ambion). Briefly, cells were disrupted in TRIzol containing 0.2 g of 0.2-mm-diameter glass beads (acid washed; Sigma) using a FastPrepH-24 instrument (MP Biomedicals), and the following extraction steps were performed according to the manufacturer’s instructions. DNase treatment was performed according to the on-column PureLink DNase treatment protocol (Life Technologies/Invitrogen). RNA was quantified on a NanoDrop ND-1000 spectrophotometer (NanoDrop Technologies, Inc.), and the quality and integrity were checked using the Experion RNA StdSens analysis kit (Bio-Rad).

The transcriptomes of the *Synechocystis* wild-type and *kpsM* mutant strains were analyzed by RNA sequencing (RNA-seq), using three biological replicates. RNA-seq data were generated by Novogene. A total amount of 3 to 5 μg RNA per sample was used as the input material for the RNA sample preparations. A detailed description of the procedure can be found in Text S1. The distribution of the identified proteins into functional categories was performed as described above.

### O_2_ evolution measurements (photosynthetic activity and respiration).

O_2_ evolution was measured using a Clark-type O_2_ electrode (Oxygraph; Hansatech Ltd.). Calibration was performed using sodium bisulfite and air-saturated water at 30°C. Assays were carried out using 1 ml of culture (previously centrifuged at 3,500 × *g* for 90 s to remove the medium/extracellular polysaccharides and resuspended in BG11 medium), at 30°C and 100 rpm. The O_2_ net evolution of cells collected in the middle of the light period of the 12-h light/12-h dark growth regimen was assessed under standard growth irradiance of 50 μE m^−2^ s^−1^. Respiration was also assessed but in samples collected in the middle of the dark period.

### Transmission electron microscopy.

Cells were fixed directly in culture medium with final concentrations of 2.5% glutaraldehyde and 2% paraformaldehyde in 0.05 M sodium cacodylate buffer (pH 7.2) (overnight), washed three times in double-strength buffer followed by postfixation with 2% osmium tetroxide in 0.1 M sodium cacodylate buffer (pH 7.2) (overnight), and washed again in the same buffer. The samples were further processed as described previously (71), except that samples were embedded in EMBed-812 resin (Electron Microscopy Sciences) and sections were examined using a JEM-1400Plus instrument (JEOL Ltd., Inc.). For peptidoglycan thickness measurements, 35 and 41 transmission electron microscopy (TEM) micrographs of the wild type and the *kpsM* mutant were used, respectively. Peptidoglycan thickness was measured in four points of the cell whenever possible.

### Statistical analysis.

Data were statistically analyzed with GraphPad Prism v5 (GraphPad Software) using analysis of variance (ANOVA), followed by Tukey’s multiple-comparison test, or using the *t* test.

### Data availability.

New RNAseq data provided in this paper have been deposited in the Gene Expression Omnibus (GEO) under accession no. GSE165073.
