# Supplementary figures and images for: Absence of KpsM (Slr0977) Impairs the Secretion of Extracellular Polymeric Substances (EPS) and Impacts Carbon Fluxes in Synechocystis sp. PCC 6803
Source: mSphere. 2021 Jan 27;6(1):e00003-21. doi: 10.1128/mSphere.00003-21 (PMC7885315; doi:10.1128/mSphere.00003-21)

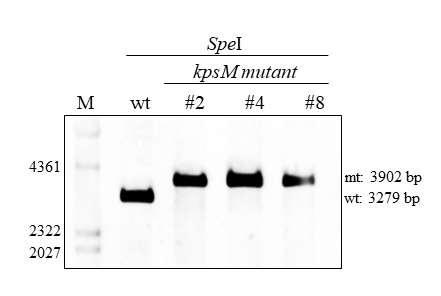

Supplement: FIG S1 [file mSphere.00003-21-sf001.tif]

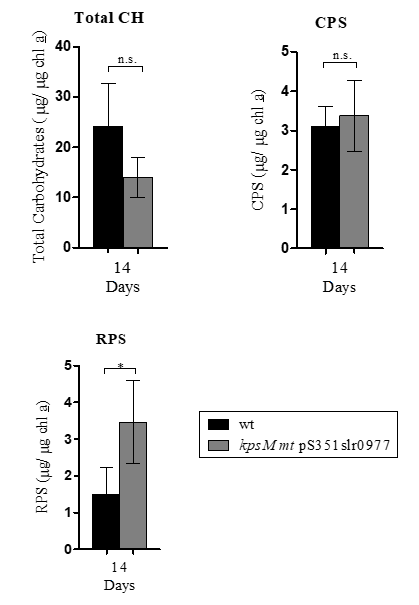

Supplement: FIG S2 [file mSphere.00003-21-sf002.tif]

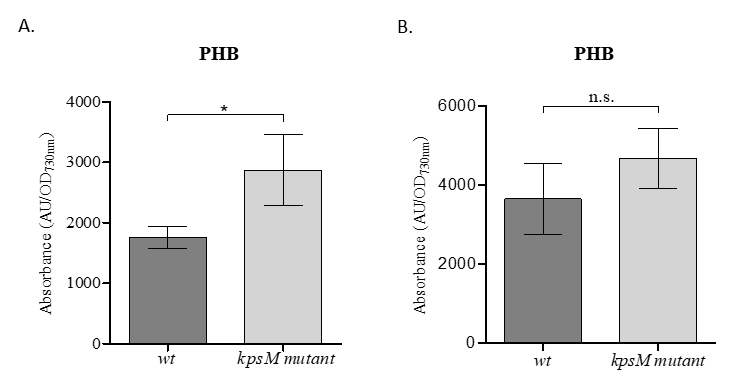

Supplement: FIG S3 [file mSphere.00003-21-sf003.tif]

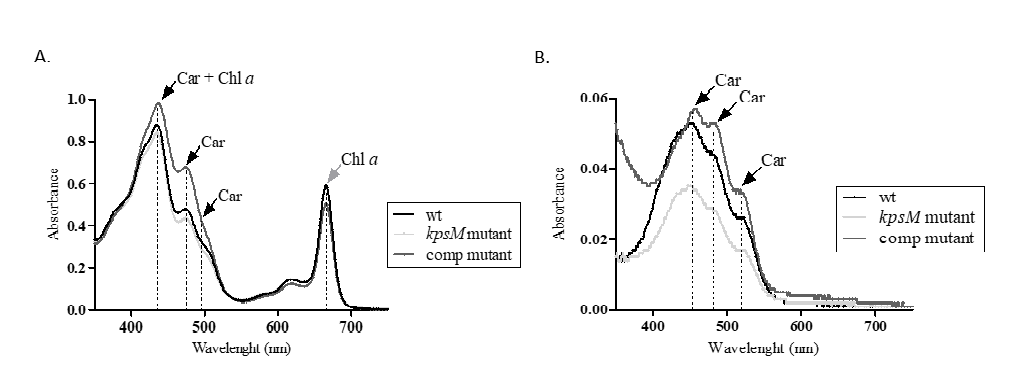

Supplement: FIG S4 [file mSphere.00003-21-sf004.tif]

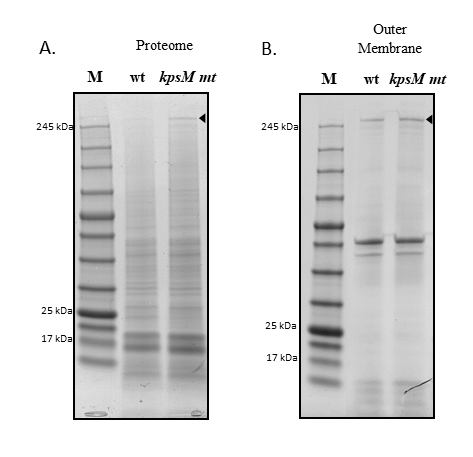

Supplement: FIG S5 [file mSphere.00003-21-sf005.tif]
