# Supplementary material for: Absence of KpsM (Slr0977) Impairs the Secretion of Extracellular Polymeric Substances (EPS) and Impacts Carbon Fluxes in Synechocystis sp. PCC 6803
Source: mSphere. 2021 Jan 27;6(1):e00003-21. doi: 10.1128/mSphere.00003-21 (PMC7885315; doi:10.1128/mSphere.00003-21)
Supplement: TABLE S1 [file mSphere.00003-21-st001.docx]

**Table S1.** Distribution by functional categories of the genes quantified in the RNAseq analysis with significant fold changes in mRNA transcript levels in *Synechocystis* *kpsM* mutant vs. wild type.

| ORF | Gene ID | Description | Fold Changes  (mt:wt) |
| --- | --- | --- | --- |
| *Photosynthesis* |  |  |  |
| *slr0148* | *-* | ferredoxin | -2,6 |
| *slr0750* | *chlN* | light-independent protochlorophyllide reductase subunit | -3,0 |
| *sll0199* | *petE* | Plastocyanin | -1,7 |
| *slr1828* | *petF* | ferredoxin | 2,1 |
| *sll0662* | *-* | ferredoxin (bacterial type ferredoxin family) | -2,0 |
| *sll1584* | *-* | ferredoxin like protein | -3,0 |
| *sll1317* | *petA* | apocytochrome f, component of cytochrome b6/f complex | -2,2 |
| *ssr2831* | *psaE* | Photosystem I reaction center subunit IV | 1,8 |
| *slr0342* | *petB* | cytochrome b6 | -1,7 |
| *sll1194* | *psbU* | photosystem II 12 kD extrinsic protein | -1,6 |
| *ssr0390* | *psaK1* | photosystem I reaction center subunit X | 1,7 |
| *ssr3451* | *psbE* | cytochrome b559 alpha subunit | -1,6 |
| *slr0150* | *petF* | ferredoxin | -1,9 |
| *slr0753* | *p* | P protein | -1,9 |
| *slr1459* | *apcF* | phycobilisome core component | -1,5 |
| *sll0550* | *Dfa1* | Diflavin flavoprotein A1 (NADH:oxygen oxidoreductase) | -1,7 |
| *sml0008* | *psaJ* | Photosystem I reaction center subunit IX | 1,7 |
| *sll0629* | *psaK* | photosystem I subunit X | 1,6 |
| *sll0427* | *psbO* | photosystem II manganese-stabilizing polypeptide | -1,5 |
| *ssl0453* | *nblA* | phycobilisome degradation protein | -1,6 |
| *ssr2049* | *bchB* | protochlorophillide reductase 57 kD subunit | -2,7 |
| *smr0004* | *psaI* | photosystem I subunit VIII | 1,6 |
| *slr2051* | *cpcG; cpcG1* | Phycobilisome rod-core linker polypeptide | 1,4 |
| *-* | *psbZ* | photosystem II | -1,5 |
| *sll1513* | *ccsA* | c-type cytochrome synthesis protein | -1,3 |
| *sll0819* | *psaF* | Photosystem I reaction center subunit III (PSI-F) | 1,4 |
| *sll1867* | *psbA3* | photosystem II D1 protein | -2,1 |
| *ssr3383* | *apcC* | Phycobilisome, allophycocyanin-associated | -1,5 |
|  |  |  |  |
| *smr0008* | *psbJ* | photosystem II PsbJ protein | -2,0 |
| *ssl0563* | *psaC* | photosystem I subunit VII | -1,3 |
| *sll1316* | *petC* | plastoquinol--plastocyanin reductase | -1,4 |
| *smr0005* | *psaM* | photosystem I PsaM subunit | 1,4 |
| *Oxidative Phosphorylation* | | | |
| *sll1324* | *atpF* | ATP synthase subunit b | 2,2 |
| *sll1323* | *atpG* | ATP synthase subunit b' | 2,2 |
| *slr1137* | *ctaD* | cytochrome c oxidase subunit I | -1,7 |
| *slr1136* | *ctaC* | cytochrome c oxidase subunit II | -1,7 |
| *slr1233* | *frdA* | Succinate dehydrogenase flavoprotein subunit | -1,4 |
| *sll0223* | *ndhB* | NAD(P)H dehydrogenase I subunit 2 | 1,5 |
| *sll1322* | *atpI* | ATP synthase subunit a | 1,7 |
| *sll1325* | *atpD* | ATP synthase d subunit | 1,8 |
| *slr1138* | *ctaE* | cytochrome c oxidase subunit III | -1,7 |
| *sll1899* | *ctaB* | cytochrome c oxidase folding protein | 1,4 |
| *sll0522* | *ndhE* | NADH dehydrogenase subunit 4L | -1,7 |
| *sll0813* | *ctaC* | cytochrome c oxidase subunit II | 1,4 |
| *slr0851* | *ndh* | NADH dehydrogenase | 1,4 |
| *Carbon Metabolism* | | | |
| *sll1776* | *deoC* | deoxyribose-phosphate aldolase | -3,5 |
| *slr0194* | *rpiA* | Ribose-5-phosphate isomerase A | -2,5 |
| *slr0985* | *rfbC* | dTDP-6-deoxy-L-mannose-dehydrogenase | 2,8 |
| *slr0983* | *rfbF* | glucose-1-phosphate cytidylyltransferase | 2,1 |
| *slr0953* | *-* | sucrose-6-phosphatase | -2,0 |
| *slr0301* | *ppsA* | phosphoenolpyruvate synthase | -2,1 |
| *slr0984* | *rfbG* | CDP-glucose 4,6-dehydratase | 2,3 |
| *slr1349* | *pgi* | glucose-6-phosphate isomerase | -1,7 |
| *slr0237* | *glgX* | glycogen operon protein; GlgX | -1,6 |
| *sll1723* | *-* | Probable glycosyltransferase | 2,5 |
| *slr1945* | *pgm* | 2,3-bisphosphoglycerate-independent phosphoglycerate mutase | 1,6 |
| *slr0288* | *glnN* | glutamine synthetase | 1,6 |
| *sll0745* | *pfkA* | phosphofructokinase | -1,6 |
| *sll1664* | *-* | probable glycosyl transferase | 1,5 |
| *sll1023* | *sucC* | succinate--CoA ligase | -1,9 |
| *sll1031* | *ccmM* | carbon dioxide concentrating mechanism protein | -1,6 |
| *slr1830* | *phbC* | polyhydroxyalkanoate synthase subunit PhaC | -1,7 |
| *slr0344* | *rbfW* | Glycosyltransferase | -2,0 |
| *slr0943* | *fda* | fructose-bisphosphate aldolase | -1,6 |
| *sll1231* | *mtfB* | Mannosyltransferase B | -1,8 |
| *slr0606* | *-* | Probable glycosyltansferase | 1,4 |
| *slr1843* | *zwf* | Glucose-6-phosphate 1-dehydrogenase | 1,4 |
| *sll1535* | *rfbP* | galactosyl-1-phosphate transferase | 1,4 |
| *slr1050* | *-* | dolichyl-phosphate-mannose-protein mannosyltransferase | -1,5 |
| *slr1513* | *-* | Part of the SbtA/B Ci uptake system | -1,6 |
| *slr2132* | *pta* | phosphotransacetylase | 1,5 |
| *slr2116* | *spsA* | spore coat polysaccharide biosynthesis protein | -1,6 |
| *slr0394* | *pgk* | phosphoglycerate kinase | 1,3 |
| *sll0920* | *ppc* | phosphoenolpyruvate carboxylase | -1,4 |
| *Motility* |  |  |  |
| *sll0043* | *-* | Cyanobacterial hybrid kinase | -3,3 |
| *sll0041* | *-* | Putative methyl-accepting chemotaxis protein | -2,1 |
| *slr1276* | *-* | type IV pilus assembly protein PilO | -1,7 |
| *slr1275* | *-* | type IV pilus assembly protein PilN | -1,8 |
| *sll1694* | *hofG* | General secretion pathway protein G | -1,5 |
| *sll1533* | *pilT* | twitching mobility protein | -1,8 |
| *sll1291* | *-* | twitching motility two-component system response regulator PilG | 2,0 |
| *slr1044* | *mcpA* | Methyl-accepting chemotaxis protein | -1,5 |
| *sll1695* | *hofG* | type IV pilus assembly protein PilA | -1,3 |
| *sll0039* | *-* | CheY subfamily | -1,3 |
| *Amino Sugar and Nucleotide Sugar Metabolism* | | | |
| *slr0017* | *murA* | UDP-N-acetylglucosamine 1-carboxyvinyltransferase | 2,8 |
| *slr1746* | *murI* | Glutamate racemase | 3,0 |
| *slr1423* | *murC* | UDP-N-acetylmuramate-alanine ligase | 2,2 |
| *Nucleotide Metabolism* | | | |
| *slr0597* | *purH* | phosphoribosyl aminoimidazole carboxy formyl formyltransferase/inosinemonophosphate cyclohydrolase; PUR-H(J) | -1,6 |
| *sll0368* | *pyrR* | Bifunctional protein PyrR [Includes: Pyrimidine operon regulatory protein; Uracil phosphoribosyltransferase (UPRTase) | 1,6 |
| *slr0520* | *purL* | phosphoribosyl formylglycinamidine synthase | 1,7 |
| *sll1823* | *purA* | Adenylosuccinate synthetase (AMPSase) (AdSS) (IMP-- aspartate ligase) | -1,4 |
| *sll0646* | *cyaA* | adenylate cyclase | -1,6 |
| *slr0379* | *-* | dTMP kinase | 1,7 |
| *Cell Envelope and Lipid Metabolism* | | | |
| *ssl1498* | *-* |  | -5,2 |
| *slr1028* | *-* | integrin alpha-subunit domain homologue | 2,0 |
| *slr0993* | *nlpD* | lipoprotein | 2,1 |
| *slr0938* | *-* | lipid II isoglutaminyl synthase | -2,1 |
| *ssl3177* | *repA* | rare lipoprotein A | 2,1 |
| *slr1744* | *amiA* | N-acetylmuramoyl-L-alanine amidase | 1,6 |
| *slr0883* | *psr* | polyisoprenyl-teichoic acid--peptidoglycan teichoic acid transferase | 1,8 |
| *sll0513* | *-* | farnesyl-diphosphate farnesyltransferase | 2,0 |
| *sll0083* | *gmhA* | Phosphoheptose isomerase | 1,6 |
| *sll1775* | *-* | non-lysosomal glucosylceramidase | -1,6 |
| *slr0488* | *-* | putative peptidoglycan lipid II flippase | 1,8 |
| *sll1522* | *pgsA* | CDP-diacylglycerol--glycerol-3-phosphate 3-phosphatidyltransferase | -1,7 |
| *slr0193* | *-* | RNA-binding protein (involved in lipid peroxidation and change of degree of lipid unsaturation) | -1,3 |
| *slr0408* | *-* | integrin alpha subunit domain homologue | 1,3 |
| *slr1677* | *-* | lipid-A-disaccharide synthase | -2,7 |
| *sll0914* | *-* | lipid metabolic process | 2,1 |
| *Protein / RNA Folding and Degradation* | | | |
| *slr0083* | *crhR; deaD* | RNA helicase CrhR (mRNA degradation) | -2,8 |
| *slr0551* | *-* | ribonuclease J | -2,0 |
| *slr1129* | *rne* | ribonuclease E | -2,1 |
| *slr0165* | *clpP3* | ATP-dependent Clp protease proteolytic subunit 3 | -2,0 |
| *sll0430* | *htpG* | Heat shock protein | -1,8 |
| *sll1679* | *hhoA* | Putative serine protease | 1,7 |
| *sll1463* | *ftsH4* | ATP-dependent zinc metalloprotease FtsH 4 | -1,5 |
| *slr0164* | *clpR* | ATP-dependent Clp protease proteolytic subunit-like | -1,6 |
| *slr2076* | *GroL1, Cpn60-1, GroEL1* | Chaperonin 1 | -1,5 |
| *sll1043* | *pnp* | polyribonucleotide nucleotidyltransferase | -1,5 |
| *ssr3307* | *Ycf47* | preprotein translocase subunit SecG | -1,3 |
| *sll0535* | *clpX* | ATP-dependent Clp protease ATP-binding subunit | -1,4 |
| *sll0416* | *GroL2, Cpn60-2, GroEL2* | Chaperonin 2 | -1,4 |
| *sll1910* | *zam* | ribonuclease R | -1,5 |
| *sll0020* | *clpC* | ATP-dependent Clp protease regulatory subunit | -1,4 |
| *Transcription* |  |  |  |
| *slr1545* | *sigG* | Sigma factor G | 2,9 |
| *sll1961* | *-* | GntR family transcriptional regulator | 2,2 |
| *sll1742* | *nusG* | transcription antitermination protein | -2,0 |
| *sll1689* | *sigE; rpoD* | Sigma factor E | 1,8 |
| *sll1818* | *rpoA* | RNA polymerase alpha subunit | -2,0 |
| *slr1912* | *-* | anti-sigma F factor antagonist | 2,1 |
| *sll1789* | *rpoC2* | RNA polymerase beta prime subunit | -1,6 |
| *slr1738* | *-* | Fur family transcriptional regulator, peroxide stress response regulator | -1,7 |
| *sll1787* | *rpoB* | RNA polymerase beta subunit | -1,8 |
| *slr0743* | *nusA* | N utilization substance protein | -1,6 |
| *sll0306* | *rpoD* | RNA polymerase sigma factor | -1,5 |
| *sll0856* | *rpoE* | RNA polymerase sigma-E factor | 1,8 |
| *sll0567* | *fur* | ferric uptake regulation protein | 1,5 |
| *slr0302* | *-* | two-component sensor activity, regulation of transcription | -1,4 |
| *sll0998* | *-* | LysR transcriptional regulator | 1,4 |
| *Translation* |  |  |  |
| *sll1799* | *rplC* | 50S ribosomal protein L3 | -2,1 |
| *sll1101* | *rpsJ* | 30S ribosomal protein S10 | -2,4 |
| *slr1795* | *msrA* | peptide methionine sulfoxide reductase | -2,2 |
| *sll1743* | *rplK* | 50S ribosomal protein L11 | -2,1 |
| *sll1866* | *-* | L-threonylcarbamoyladenylate synthase | -2,2 |
| *ssl1426* | *Rpl35* | 50S ribosomal protein L35 | -1,6 |
| *sll0947* | *Hpf; lrtA* | Ribosome hibernation promotion factor (HPF) (Light-repressed protein A) | -1,6 |
| *-* | *rpmG* | large subunit ribosomal protein L33 | -1,7 |
| *sll1261* | *tsf* | Elongation factor Ts (EF-Ts) | -1,7 |
| *sll1816* | *rpsM; rps13* | 30S ribosomal protein S13 | -1,5 |
| *slr1031* | *tyrS* | tyrosyl tRNA synthetase | -1,8 |
| *ssr1399* | *rpsR; rps18* | 30S ribosomal protein S18 | -1,6 |
| *slr1550* | *lysS* | lysyl-tRNA synthetase | -1,9 |
| *slr0923* | *-* | Probable 30S ribosomal protein PSRP-3 (Ycf65-like protein) | -1,5 |
| *sll1810* | *rplF; rpl6* | 50S ribosomal protein L6 | 1,5 |
| *sll1821* | *rplM; rpl13* | 50S ribosomal protein L13 | -1,6 |
| *ssr0482* | *Rps16* | 30S ribosomal protein S16 | -1,4 |
| *sll0767* | *rplT; rpl20* | 50S ribosomal protein L20 | -1,5 |
| *sll1817* | *rpsK; rps11* | 30S ribosomal protein S11 | -1,5 |
| *sll1260* | *rpsB; rps2* | 30S ribosomal protein S2 | -1,5 |
| *sll1822* | *rpsI; rps9* | 30S ribosomal protein S9 | -1,4 |
| *sll0495* | *asnS* | asparaginyl-tRNA synthetase | 1,4 |
| *ssr1736* | *Rps32* | 50S ribosomal protein L32 | -1,5 |
| *sll1097* | *rpsG; rps7* | 30S ribosomal protein S7 | -1,5 |
| *slr0120* | *-* | tRNA/rRNA methyltransferase | 2,0 |
| *sll1740* | *rplS; rpl19* | 50S ribosomal protein L19 | -1,3 |
| *slr1115* | *-* | tRNA (cmo5U34)-methyltransferase | -1,7 |
| *slr0033* | *-* | aspartyl-tRNA(Asn)/glutamyl-tRNA(Gln) amidotransferase subunit C | -1,3 |
| *Cofactors and Vitamins Metabolism* | | | |
| *slr1434* | *pntB* | pyridine nucleotide transhydrogenase beta subunit | -2,9 |
| *slr0536* | *hemE* | uroporphyrinogen decarboxylase | -1,9 |
| *slr1916* | *-* | Probable esterase | -2,1 |
| *slr0506* | *Por; pcr* | Light-dependent protochlorophyllide reductase (PCR) (NADPHprotochlorophyllide oxidoreductase) (LPOR) (POR) | -1,8 |
| *sll0898* | *-* | thiamine phosphate phosphatase | -1,9 |
| *slr1882* | *-* | riboflavin kinase | -1,9 |
| *sll1876* | *hemN* | oxygen independent coprophorphyrinogen III oxidase | -1,8 |
| *slr1808* | *hemA* | Glutamyl-tRNA reductase (GluTR) | -1,6 |
| *slr1518* | *menA* | 1,4-dihydroxy-2-naphtoic acid prenyltransferase | 1,7 |
| *sll1282* | *ribH* | riboflavin synthase beta subunit | -1,7 |
| *slr0902* | *moaC* | molybdenum cofactor biosynthesis protein C | -2,2 |
| *sll0166* | *hemD* | uroporphyrin-III synthase | -1,4 |
| *slr0749* | *chlL* | light-independent protochlorophyllide reductase iron protein subunit | -1,6 |
| *slr0711* | *-* | 7-cyano-7-deazaguanine reductase | -1,5 |
| *slr0553* | *-* | dephospho-CoA kinase | -1,7 |
| *slr0901* | *moaA* | molybdenum cofactor biosynthesis protein A | -1,7 |
| *slr0427* | *-* | nicotinamide-nucleotide amidase | -1,7 |
| *slr1784* | *bvdR* | biliverdin reductase | -1,7 |
| *sll0603* | *menD* | 2-succinyl-6-hydroxy-2,4-cyclohexadiene-1-carboxylate synthase | -1,6 |
| *slr1780* | *-* | Ycf54-like protein | -1,5 |
| *sll1415* | *-* | NAD+ kinase | -1,4 |
| *slr0239* | *cbiF* | precorrin methylase | -1,6 |
| *DNA Replication and Repair* | | | |
| *sll1772* | *mutS* | DNA mismatch repair protein MutS | -2,8 |
| *slr1130* | *rhnB* | ribonuclease HII | -2,4 |
| *sll1629* | *phr* | DNA photolyase | -2,1 |
| *slr1822* | *nth* | endonuclease III | -2,0 |
| *sll1429* | *-* | similar to archaeal holliday junction resolvase and Mrr protein | 1,9 |
| *slr1803* | *mbpA* | adenine-specific DNA metylase | -2,0 |
| *slr0965* | *dnaN* | DNA polymerase III beta subunit | -2,0 |
| *sll0729* | *-* | modification methylase | -1,9 |
| *sll1143* | *uvrD* | ATP-dependent DNA helicase | -1,5 |
| *slr1048* | *-* | DNA repair protein SbcC/Rad50 | -1,7 |
| *sll0569* | *recA* | Protein RecA (Recombinase A) | 1,3 |
| *slr0020* | *recG* | DNA recombinase | -1,3 |
| *slr0181* | *-* | DNA repair protein RecO | 1,3 |
| *sll1099* | *tufA* | elongation factor Tu | -1,4 |
| *sll0270* | *priA* | primosomal protein N' | -1,6 |
| *Cell Division* |  |  |  |
| *slr1604* | *-* | cell division protein FtsH | -2,1 |
| *slr2073* | *-* | cell division inhibitor SepF | 1,6 |
| *slr0228* | *ftsH* | cell division protein | -1,5 |
| *slr1267* | *mrdB* | cell division protein FtsW | 1,5 |
| *sll1632* | *-* | cell division protein FtsQ | 1,3 |
| *Amino Acid Metabolism* | | | |
| *slr0055* | *trpG* | anthranilate synthase component II | -2,0 |
| *slr0543* | *trpB* | tryptophan synthase beta subunit | -1,5 |
| *sll1760* | *thrB* | homoserine kinase | 1,9 |
| *slr0528* | *murE* | UDP-MurNac-tripeptide synthetase | 1,7 |
| *sll0228* | *-* | arginase | -1,8 |
| *slr0827* | *alr* | alanine racemase | 1,4 |
| *slr0596* | *-* | creatinine amidohydrolase | -1,6 |
| *sll1561* | *putA* | delta-1-pyrroline-5-carboxylate dehydrogenase | -1,4 |
| *sll0892* | *panD* | aspartate 1-decarboxylase | -1,5 |
| *slr1705* | *aspA* | aspartoacylase | -1,6 |
| *slr0689* | *-* | L-aspartate semialdehyde sulfurtransferase | 1,4 |
| *sll0934* | *ccmA* | Carboxysome formation protein | -1,4 |
| *sll0006* | *aspC* | aspartate aminotransferase | -1,5 |
| *slr0549* | *asd* | aspartate beta-semialdehyde dehydrogenese | -1,3 |
| *sll0402* | *aspC* | aspartate aminotransferase | -1,4 |
| *slr2079* | *-* | glutaminase | 1,6 |
| *Other Signalling and Cellular Processes* | | | |
| *sll0042* | *tar* | methyl-accepting chemotaxis protein II | -3,8 |
| *slr0121* | *-* | beta-lactamase class A | 3,2 |
| *slr0323* | *ams1* | α-Mannosidase | -2,5 |
| *slr0474* | *Rcp1* | response regulator | -2,8 |
| *slr1963* | *-* | Orange carotenoid-binding protein (OCP) | -2,1 |
| *sll1566* | *otsA* | glucosylglycerol-phosphate synthase | 2,1 |
| *slr1805* | *-* | two-component sensor histidine kinase | 2,5 |
| *ssl0707* | *glnB* | nitrogen regulatory protein P-II | 1,9 |
| *slr1971* | *-* | Zn-dependent protease | 2,1 |
| *slr0242* | *bcp* | bacterioferritin comigratory protein | -1,9 |
| *slr2089* | *shc* | squalene-hopene-cyclase | -1,8 |
| *sll0005* | *-* | aarF domain-containing kinase | -1,9 |
| *slr1269* | *ggt* | gamma-glutamyltranspeptidase | 2,1 |
| *sll0080* | *argC* | N-acetyl-gamma-glutamyl-phosphate reductase | 1,8 |
| *sll1226* | *hoxH* | Oxireductase (hydrogen as donor), NIFe bidirectional hydrogenase | -1,8 |
| *slr0926* | *ubiA* | 4-hydroxybenzoate-octaprenyl transferase | -1,9 |
| *sll1678* | *-* | spore maturation protein A | 2,5 |
| *sll1786* | *tatD* | DNase family protein | -1,7 |
| *slr1472* | *-* | spoIIIJ-associated protein | -2,7 |
| *sll1454* | *narB* | nitrate reductase | -2,3 |
| *sll1933* | *dnaJ* | DnaJ protein | -2,1 |
| *slr1414* | *-* | sensory transduction histidine kinase | -2,5 |
| *sll1677* |  | spore maturation protein B | 2,7 |
| *slr1348* | *cysE* | serine acetyltransferase | -1,8 |
| *slr0841* | *-* | heat shock protein HslJ | 1,7 |
| *slr1950* | *-* | cation-transporting ATPase | -1,6 |
| *sll1314* | *dctP* | C4-dicarboxylase binding protein | 2,1 |
| *slr0550* | *dapA* | dihydrodipicolinate synthase | -1,8 |
| *sll1223* | *hoxU* | hydrogenase subunit | -1,6 |
| *slr0077* | *Csd; sufS* | Probable cysteine desulfurase | -1,9 |
| *ssl3335* | *secE* | secretory protein; SecE | -1,6 |
| *slr0328* | *wzb* | low molecular weight protein-tyrosine-phosphatase | -2,7 |
| *ssl2923* | *vapC* | virulence associated protein C | -3,0 |
| *slr0742* | *-* | ribosome maturation factor RimP | -1,7 |
| *slr0423* | *rlpA* | rare lipoprotein A | 2,0 |
| *sll1783* | *-* | Monooxygenase (associated to polysaccharide processing) | 2,0 |
| *sll1068* | *acp* | acyl carrier protein | -2,2 |
| *slr1849* | *merA* | mercuric reductase | -2,1 |
| *slr0605* | *-* | Oxireductase (quinone as aceptor), putative glutathione S transferase | -1,6 |
| *sll1515* | *gifB* | glutamine synthetase inactivating factor IF17 | -2,1 |
| *slr1924* | *-* | D-alanyl-D-alanine carboxypeptidase | -1,6 |
| *slr0484* | *-* | sensory transduction histidine kinase | 1,5 |
| *sll0474* | *-* | sensory transduction histidine kinase | -2,0 |
| *sll2009* | *-* | processing protease | -1,8 |
| *sll1224* | *hoxY* | hydrogenase small subunit | -1,6 |
| *sll1825* | *-* | aklaviketone reductase | 1,6 |
| *slr1400* | *-* | hybrid sensory kinase | 1,5 |
| *sll1771* | *pphA* | protein serin-threonin phosphatase | -1,5 |
| *sll0222* | *phoA* | Alkaline phosphatase | -1,6 |
| *sll0337* | *sphS* | regulation of the phosphate regulon | 1,5 |
| *slr1728* | *kdpA* | otassium-transporting ATPase A chain | 2,1 |
| *slr1641* | *clpB* | ClpB protein | -1,4 |
| *slr1594* | *-* | PatA subfamily | 1,8 |
| *sll1462* | *hypE* | hydrogenase expression/formation protein | -1,6 |
| *sll1626* | *lexA* | Transcription regulator LexA | 1,6 |
| *slr1207* | *-* | HlyD family secretion protein | 1,5 |
| *slr1639* | *smpB* | SsrA-binding protein | -1,6 |
| *sll1353* | *-* | sensory transduction histidine kinase | -1,6 |
| *sll1283* | *spoIID* | sporulation protein | 1,5 |
| *slr1668* | *-* | fimbrial chaperone protein | -1,7 |
| *sll1124* | *-* | sensory transduction histidine kinase | -1,5 |
| *slr1516* | *sobB* | superoxide dismutase | -1,4 |
| *sll0659* | *-* | lycopene cyclase CruP | -1,7 |
| *slr0687* | *pleD* | PleD gene product | 1,8 |
| *slr0473* | *phy* | phytochrome | -1,5 |
| *slr0457* | *truB* | tRNA pseudouridine 55 synthase tRNA pseudouridine 55 synthase | -2,0 |
| *slr2135* | *hupE* | hydrogenase accessory protein | 1,7 |
| *sll1666* | *dnaJ* | DnaJ protein | 1,5 |
| *sll1187* | *lgt* | prolipoprotein diacylglyceryl transferase | -1,4 |
| *sll0247* | *isiA* | iron-stress chlorophyll-binding protein | 1,7 |
| *ssl2922* | *vapB* | virulence associated protein B | -3,1 |
| *slr0801* | *-* | putative flavoprotein involved in K+ transport | 1,7 |
| *slr0509* | *-* | alkaline phosphatase like protein | 1,5 |
| *sll1770* | *spkI* | protein kinase activity, protein phosphorylation | -1,4 |
| *slr2123* | *-* | D-isomer specific 2-hydroxyacid dehydrogenase family | -1,5 |
| *sll0330* | *fabG* | 3-ketoacyl-acyl carrier protein reductase | 1,5 |
| *sll1929* | *comEc* | competence protein ComEC | 1,6 |
| *sll1475* | *-* | sensory transduction histidine kinase | -1,7 |
| *slr0701* | *merR* | mercuric resistance operon regulatory protein | 1,9 |
| *sll0782* | *-* | putative protein kinase | 1,8 |
| *sll0260* | *-* | putative hemolysin | 1,4 |
| *slr1760* | *-* | regulatory components of sensory transduction system | 1,7 |
| *slr1983* | *-* | two-component system, response regulator | -1,5 |
| *slr0095* | *-* | O-methyltransferase | -1,6 |
| *sll0410* | *-* | acyl-CoA thioester hydrolase | -1,4 |
| *slr0079* | *gspE* | general secretion pathway protein E | -1,3 |
| *sll1394* | *msrA* | peptide methionine sulfoxide reductase | -1,5 |
| *sll1468* | *bhy* | b-carotene hydroxylase | 1,4 |
| *slr0348* | *-* | 4-hydroxy-3-methylbut-2-en-1-yl diphosphate reductase | 1,3 |
| *slr0659* | *prlC* | oligopeptidase A | -1,3 |
| *Transporters* |  |  |  |
| *sll1762* | *-* | ABC amino acid transporter | 1,5 |
| *slr1227* | *-* | chloroplast import-associated channel IAP75 | 2,2 |
| *sll0224* | *-* | - | 3,0 |
| *sll0384* | *-* | cobalt/nickel transport system permease | -3,2 |
| *sll0385* | *cbiO* | ATP-binding protein of ABC transporter | -2,6 |
| *slr0797* | *-* | cation-transporting ATPase | -2,5 |
| *slr0610* | *-* | ABC-2 type transport system permease | -2,8 |
| *slr1319* | *-* | iron-uptake system ATP-binding protein | 2,5 |
| *sll0923* | *epsB; wzc* | exopolysaccharide export protein | 2,0 |
| *sll1087* | *-* | sodium-coupled permease | 2,0 |
| *sll0383* | *cbiM* | cobalt/nickel transport system permease | -2,5 |
| *sll0108* | *-* | putative ammonium transporter | 2,0 |
| *sll1581* | *gumB* | polysaccharide biosynthesis/export | 1,8 |
| *slr1229* | *-* | sulfate permease | -2,7 |
| *sll0382* | *-* | \|  \| nickel transport protein \| \| --- \| --- \| | -1,9 |
| *slr0982* | *rfbB* | lipopolysaccharide transport system ATP-binding protein | 1,9 |
| *sll1024* | *-* | ion channel-forming bestrophin family protein | -2,5 |
| *sll1600* | *mntB* | Mn transporter | -1,9 |
| *slr1113* | *-* | ABC transporter | 1,6 |
| *slr2107* | *-* | probable polysaccharide ABC transporter permease protein | -2,5 |
| *slr0773* | *-* | trk system potassium uptake protein | -1,8 |
| *sll0679* | *sphX* | phosphate transport system substrate-binding protein | 2,5 |
| *sll1864* | *-* | chloride channel protein | -1,7 |
| *sll1482* | *-* | ABC-transporter DevC homologue | -1,9 |
| *slr0677* | *exbB* | biopolymer transport ExbB protein | 1,5 |
| *slr0369* | *-* | cation or drug efflux system protein | 1,6 |
| *slr1295* | *sufA* | iron transport protein | 1,8 |
| *sll1017* | *-* | putative ammonium transporter | 2,4 |
| *sll1374* | *melB* | melibiose carrier protein | 1,6 |
| *sll0671* | *-* | magnesium transporter | 1,7 |
| *slr1457* | *chrA* | chromate transport protein | -1,8 |
| *sll1845* | *-* | translocator protein | 1,7 |
| *sll1406* | *fhuA* | ferrichrome-iron receptor | 1,7 |
| *slr0354* | *-* | ABC transporter | 1,9 |
| *slr0044* | *nrtD* | nitrate transport protein | -2,5 |
| *slr1270* | *tolC* | outer membrane factor | 1,4 |
| *slr0964* | *-* | high-affinity iron transporter | -1,6 |
| *sll0985* | *-* | moderate conductance mechanosensitive channel | -1,6 |
| *sll0616* | *secA* | Protein translocase subunit SecA | -1,4 |
| *slr1200* | *livH* | high-affinity branched-chain amino acid transport protein | 1,7 |
| *slr0075* | *Ycf16* | ABC transporter subunit | -1,6 |
| *slr1201* | *-* | urea transport system permease protein | 1,8 |
| *sll0834* | *-* | low affinity sulfate transporter | -1,5 |
| *sll1586* | *-* | translocation and assembly module TamB | 1,4 |
| *slr2019* | *-* | ABC transporter | -1,4 |
| *slr2131* | *-* | cation or drug efflux system protein | 1,4 |
| *slr0305* | *-* | Putative membrane protein | -2,0 |
| *sll0374* | *brag; livF* | High-affinity branched-chain amino acid transport ATP-binding protein | 1,7 |
| *slr1647* | *-* | putative ABC transport system permease protein | -1,7 |
| *sll0672* | *-* | cation-transporting ATPase | -1,4 |
| *slr0625* | *-* | glutamate:Na+ symporter | -1,5 |
| *slr0341* | *-* | polar amino acid transport system substrate-binding protein | 1,7 |
| *slr0513* | *futA* | Iron uptake (photosystem II protection from ROS) | 1,5 |
| *sll1053* | *-* | Putative periplasmic adaptor protein (AcrA-like) | 1,6 |
| *slr1454* | *cysW* | sulfate transport system permease protein | -2,8 |
| *Hypothetical* |  |  |  |
| *slr0554* | *-* | - | 2,7 |
| *slr1610* | *-* | putative C-3 methyl transferase | 2,9 |
| *sll1464* | *-* | - | -2,5 |
| *sll1188* | *-* | - | -2,7 |
| *slr1152* | *-* | - | 1,8 |
| *sll1505* | *-* | - | -4,6 |
| *slr1542* | *-* | 2-C-methyl-D-erythritol 2,4-cyclodiphosphate synthase | -2,7 |
| *slr0967* | *-* | - | 2,1 |
| *slr1593* | *-* | - | 2,8 |
| *slr1649* | *-* | - | -2,0 |
| *slr1104* | *-* | - | -1,7 |
| *slr0483* | *-* | - | 1,7 |
| *slr0383* | *-* | - | 2,8 |
| *slr0755* | *-* | - | -1,8 |
| *slr0769* | *-* | - | 1,6 |
| *sll0189* | *-* | putative endonuclease | -1,9 |
| *sll0168* | *-* | - | -1,7 |
| *sll0545* | *-* | - | -1,9 |
| *sll0154* | *-* | hypothetical 35.6 kD protein | 1,5 |
| *sll0686* | *-* | - | 2,1 |
| *sll1526* | *-* | - | 1,7 |
| *ssl0331* | *-* | - | -1,9 |
| *sll1355* | *-* | - | 1,5 |
| *sll1680* | *-* | peptide-methionine (R)-S-oxide reductase | -1,5 |
| *slr0053* | *-* | probable rRNA maturation factor | -1,6 |
| *slr0284* | *-* | putative membrane protein | 1,6 |
| *slr1241* | *-* | - | -2,1 |
| *sll1232* | *-* | - | -2,3 |
| *sll1103* | *-* | - | 1,6 |
| *slr1851* | *-* | - | 1,4 |
| *slr0359* | *-* | - | -1,5 |
| *slr1102* | *-* | - | -1,4 |
| *sll1433* | *-* | - | -1,6 |
| *sll1738* | *-* | - | -1,4 |
| *sll1924* | *-* | - | 1,5 |
| *slr0581* | *-* | - | 1,7 |
| *slr0021* | *-* | putative protease | -1,6 |
| *slr0784* | *-* | - | 1,7 |
| *slr1261* | *-* | - | -1,5 |
| *sll0141* | *-* | - | 1,5 |
| *sll1693* | *-* | - | -1,4 |
| *slr1692* | *-* | - | -1,5 |
| *sll0297* | *-* | - | -1,4 |
| *sll1509* | *ycf20* | - | 1,6 |
| *sll1254* | *-* | Hemolysin-like | 1,4 |
| *sll0217* | *-* | potential FMN-protein | 1,4 |
| *ssr2998* | *-* | - | -1,4 |
| *sll0424* | *-* | - | -1,5 |
| *sll0036* | *-* | - | 1,4 |
| *slr0076* | *-* | Fe-S cluster assembly protein SufD | -1,4 |
| *sll0536* | *-* | - | 1,6 |
| *slr0404* | *-* | - | -1,3 |
| *sll0183* | *-* | - | 1,4 |
| *sll1504* | *-* | - | -1,8 |
| *sll1534* | *-* | - | -1,4 |
| *Unknown* |  |  |  |
| *slr1546* | *-* | Possible anti-sigma factor sigG | 3,9 |
| *slr1816* | *-* | - | -2,9 |
| *slr1218* | *-* | - | 4,3 |
| *slr1940* |  | Protein putatively involved in extracellular connection structures | 2,7 |
| *slr1547* | *-* | - | 2,8 |
| *ssr3465* | *-* | - | 2,4 |
| *sll1102* | *-* | - | 3,4 |
| *sll1722* | *-* | - | 3,1 |
| *slr0358* | *-* | - | 2,5 |
| *slr1772* | *-* | - | -2,5 |
| *slr0243* | *-* | - | -3,2 |
| *sll0781* | *-* | - | 2,5 |
| *slr1771* | *-* | - | 2,2 |
| *ssr2554* | *-* | - | 2,3 |
| *slr0976* | *-* | - | 2,1 |
| *sll1265* | *-* | - | 1,9 |
| *slr1178* | *-* | - | 2,4 |
| *sll1837* | *-* | Periplasmic protein | 2,1 |
| *ssr1853* | *-* | - | 2,3 |
| *sll1086* | *-* | - | 2,4 |
| *slr1966* | *-* | - | 2,4 |
| *slr1169* | *-* | - | -3,3 |
| *sll1834* | *-* | - | 2,1 |
| *slr1815* | *-* | - | -2,2 |
| *slr0334* | *-* | - | -2,7 |
| *sll0710* | *-* | - | -2,3 |
| *slr0061* | *-* | - | -3,9 |
| *slr1236* | *-* | - | 1,8 |
| *slr0702* | *-* | - | 2,4 |
| *slr0376* | *-* | Encoding gene is part of responsive operon to stress | 2,0 |
| *sll0314* |  | Lipoprotein (signaling) | 1,8 |
| *sll1696* | *-* | - | -2,6 |
| *sll1488* | *-* | - | 1,8 |
| *slr2126* | *-* | - | -2,4 |
| *sll1550* | *-* | - | 2,1 |
| *slr0373* | *-* | - | 1,8 |
| *slr1258* | *-* | - | 1,8 |
| *sll0983* | *-* | - | -2,0 |
| *slr0981* | *-* | - | 1,7 |
| *sll1507* | *-* | - | 1,7 |
| *slr0333* | *-* | - | -2,2 |
| *ssr3000* | *-* | - | -2,4 |
| *sll0031* | *-* | - | 2,0 |
| *slr0149* | *-* | - | -2,0 |
| *sll0862* | *-* | - | -1,7 |
| *slr1990* | *-* | - | -1,9 |
| *ssl3291* | *-* | - | 1,9 |
| *slr2052* | *-* | - | 2,3 |
| *slr0145* | *-* | - | -2,2 |
| *slr1262* | *-* | - | -1,9 |
| *sll0044* | *-* | - | -3,0 |
| *slr1415* | *-* | - | -2,0 |
| *slr1601* | *-* | - | 1,8 |
| *slr1406* | *-* | - | 1,8 |
| *sll0630* | *-* | - | 1,7 |
| *sll1378* | *-* | - | -2,0 |
| *slr1681* | *-* | - | 1,9 |
| *sll1766* | *-* | - | -1,9 |
| *sll0180* | *-* | - | 1,6 |
| *slr0505* | *-* | - | -2,1 |
| *slr1927* | *-* | - | -1,7 |
| *sll0178* | *-* | - | -1,7 |
| *slr2103* | *-* | - | -2,1 |
| *slr1590* | *-* | - | -1,6 |
| *slr1442* | *-* | - | 1,6 |
| *slr1222* | *-* | - | -2,0 |
| *sll0350* | *-* | - | 2,0 |
| *sll0072* | *-* | - | -2,4 |
| *sll1333* | *-* | - | 2,0 |
| *sll1336* | *-* | - | -1,5 |
| *ssr0336* | *-* | - | -2,1 |
| *slr0397* | *-* | - | 2,0 |
| *slr0287* | *-* | - | -1,8 |
| *slr0731* | *-* | - | -1,8 |
| *sll0756* | *-* | - | -1,7 |
| *sll0846* | *-* | - | -1,5 |
| *slr1413* | *-* | - | -1,5 |
| *sll1757* | *-* | - | -2,0 |
| *slr1866* | *-* | - | -2,1 |
| *slr1391* | *-* | - | -2,4 |
| *slr0338* | *-* | - | 1,6 |
| *sll1892* | *-* | - | 1,5 |
| *slr1240* | *-* | - | -2,0 |
| *slr1378* | *-* | - | -1,5 |
| *slr1634* | *-* | - | 1,6 |
| *slr0592* | *-* | - | 1,8 |
| *slr1173* | *-* | - | 1,5 |
| *ssr2781* | *-* | - | -2,6 |
| *slr1541* | *-* | - | -1,9 |
| *slr1928* | *-* | - | -2,1 |
| *sll0253* | *-* | - | -1,5 |
| *slr0060* | *-* | - | -2,1 |
| *sll1036* | *-* | - | -1,7 |
| *sll1222* | *-* | - | -1,6 |
| *slr1852* | *-* | - | 1,7 |
| *sll1009* | *frpC* | Protein with calcium ion binding motifs (Ironregulated protein) | 1,4 |
| *sll1543* | *-* | - | 1,4 |
| *slr0978* | *-* | - | 1,5 |
| *slr1263* | *-* | - | 1,6 |
| *slr0300* | *-* | - | -1,7 |
| *sll1873* | *-* | - | -1,4 |
| *sll0319* | *-* | - | 1,7 |
| *slr1708* | *-* | lysostaphin | 1,5 |
| *sll1634* | *-* | - | 2,0 |
| *slr0643* | *-* | - | -1,6 |
| *slr0238* | *-* | - | -1,8 |
| *sll0839* | *-* | - | -2,2 |
| *ssr1600* | *-* | - | 1,5 |
| *sll1752* | *-* | - | 1,7 |
| *sll0933* | *-* | - | -1,5 |
| *sll0266* | *-* | - | -1,6 |
| *sll1359* | *-* | - | -1,8 |
| *sll0293* | *-* | - | 1,7 |
| *sll0943* | *-* | - | 2,2 |
| *ssr3304* | *-* | - | 1,6 |
| *slr1946* | *-* | - | -1,4 |
| *sll0225* | *-* | - | 2,2 |
| *sll1917* | *-* | oxygen-independent coproporphyrinogen-III oxidase-like protein | -1,9 |
| *slr0374* | *-* | Possible aaa protease | 1,5 |
| *slr1230* | *-* | universal stress protein Slr1230-like | -1,9 |
| *sll1621* | *-* | Putative peroxiredoxin | -1,4 |
| *ssr2153* | *-* | - | 1,6 |
| *slr1576* | *-* | - | 1,8 |
| *sll1119* | *-* | - | 1,7 |
| *sll0264* | *-* | - | 1,4 |
| *sll0863* | *-* | - | -2,2 |
| *slr0244* | *-* | - | -1,4 |
| *slr2018* | *-* | - | -1,5 |
| *slr1638* | *-* | - | -1,5 |
| *slr0889* | *-* | uncharacterized protein slr0889 isoform X1 | -1,6 |
| *sll0181* | *-* | - | -1,8 |
| *slr0668* | *-* | - | -1,9 |
| *sll0783* | *-* | - | 2,3 |
| *sll0556* | *-* | - | -1,4 |
| *slr0819* | *int* | apolipoprotein N-acyltransferase | -1,5 |
| *slr0888* | *-* | - | -1,5 |
| *sll0294* | *-* | - | 1,8 |
| *slr0700* | *-* | - | -2,0 |
| *ssr2315* | *-* | - | -1,8 |
| *slr2124* | *-* | short-chain alcohol dehydrogenase family | -1,6 |
| *slr1958* | *-* | - | -1,5 |
| *sll0447* | *-* | - | -1,9 |
| *sll0670* | *-* | - | 1,7 |
| *slr2027* | *-* | - | -1,5 |
| *slr1770* | *-* | - | 1,5 |
| *ssr3570* | *-* | - | -1,8 |
| *sll1751* | *-* | - | -1,9 |
| *sll0008* | *-* | - | -1,6 |
| *slr0552* | *-* | - | -1,5 |
| *sll1913* | *-* | - | -1,7 |
| *sll1049* | *-* | - | 1,5 |
| *sll1885* | *-* | - | 1,5 |
| *ssl1533* | *-* | - | 1,4 |
| *sll1959* | *suhB* | extragenic suppressor | -1,8 |
| *ssl1046* | *-* | - | 1,8 |
| *ssl2138* | *-* | - | -2,0 |
| *ssl2920* | *-* | - | -3,2 |
| *sll1162* | *-* | - | -2,5 |
| *slr0957* | *-* | - | 1,4 |
| *slr0109* | *-* | - | -2,0 |
| *sll1106* | *-* | - | -1,4 |
| *sll1131* | *-* | - | 1,7 |
| *slr0151* | *-* | - | -1,4 |
| *slr2117* | *-* | - | -2,1 |
| *sll1681* | *-* | - | -1,5 |
| *sll0160* | *-* | - | -1,6 |
| *ssr3467* | *-* | - | 1,6 |
| *slr1926* | *-* | - | -1,3 |
| *sll0503* | *-* | - | -1,6 |
| *sll0494* | *-* | - | -1,7 |
| *slr0377* | *-* | - | 1,4 |
| *sll0543* | *-* | - | 1,5 |
| *slr1303* | *-* | - | -1,8 |
| *slr0007* | *-* | - | -1,4 |
| *slr1076* | *-* | - | 1,4 |
| *slr1095* | *-* | - | 1,5 |
| *slr1148* | *-* | - | -2,1 |
| *sll1060* | *-* | Membrane protein (UPF0182 protein) | -1,5 |
| *slr0587* | *-* | - | -1,5 |
| *sll0944* | *-* | - | 1,4 |
| *ssr3129* | *-* | - | 1,4 |
| *sll0740* | *-* | - | 1,3 |
| *sll1516* | *-* | - | -1,4 |
| *slr0038* | *-* | - | 1,4 |
| *slr1142* | *-* | - | 1,5 |
| *slr1110* | *-* | - | -1,7 |
| *slr1484* | *-* | - | 2,8 |
| *slr1612* | *-* | - | 1,4 |
| *sll0471* | *-* | - | -1,6 |
| *sll1726* | *-* | - | 1,9 |
| *sll0736* | *-* | - | -1,9 |
| *sll1396* | *-* | - | 1,4 |
| *sll0442* | *-* | - | -1,8 |
| *slr0309* | *-* | P-methylase | 1,4 |
| *sll0887* | *-* | - | -1,4 |
| *sll2003* | *-* | - | 1,5 |
| *slr2127* | *-* | - | -1,6 |
| *sll1334* | *-* | - | 1,4 |
| *sll0243* | *-* | - | -1,3 |
| *sll1251* | *-* | - | 1,5 |
| *slr0363* | *-* | - | 1,7 |
| *ssl1263* | *-* | - | -1,4 |
| *slr0147* | *-* | - | -1,5 |
| *sll0525* | *-* | - | -1,5 |
| *sll1832* | *-* | - | 1,5 |
| *sll1217* | *-* | - | 1,5 |
| *slr0119* | *brkB* | serum resistance locus | -1,9 |
| *ssl2245* | *-* | - | 1,4 |
| *slr0666* | *-* | - | 1,3 |
| *sll0488* | *-* | - | 1,4 |
| *sll0481* | *-* | - | 1,6 |
| *ssr2087* | *-* | - | 1,6 |
| *slr1081* | *-* | - | -1,4 |
| *slr1591* | *-* | - | -1,6 |
| *sll1939* | *-* | - | 1,4 |
| *slr0656* | *-* | - | -1,3 |
| *slr2141* | *-* | - | 1,5 |
| *sll0644* | *-* | esterase | 1,5 |
| *sll1089* | *-* | - | 1,4 |
| *sll0066* | *-* | - | -1,6 |
| *slr1778* | *-* | - | -1,5 |
| *sll0737* | *-* | - | -1,4 |
| *sll1159* | *-* | - | 1,5 |
| *slr1087* | *-* | - | 1,4 |
| *slr0386* | *-* | - | 1,4 |
| *sll2015* | *-* | - | 1,4 |
| *slr1056* | *-* | - | -1,4 |
| *slr0351* | *-* | - | 1,4 |
| *sll0446* | *-* | - | -1,4 |
