# Supplementary material for: Absence of KpsM (Slr0977) Impairs the Secretion of Extracellular Polymeric Substances (EPS) and Impacts Carbon Fluxes in Synechocystis sp. PCC 6803
Source: mSphere. 2021 Jan 27;6(1):e00003-21. doi: 10.1128/mSphere.00003-21 (PMC7885315; doi:10.1128/mSphere.00003-21)
Supplement: TABLE S2 [file mSphere.00003-21-st002.docx]

**Table S2.** Distribution by functional categories of the proteins quantified in the iTRAQ analysis with significant fold changes in *Synechocystis kpsM* mutant vs. wild type.

| Protein Name | Uniprot ID | Description | Fold Change  (mt:wt) |
| --- | --- | --- | --- |
| *Photosynthesis* |  |  |  |
| Sll1796; PetJ | P46445 | Cytochrome c6 (Cytochrome c553) | 3,5 |
| Ssl0020; Fed; PetF | P27320 | Ferredoxin-1 (Ferredoxin I) | 1,4 |
| Slr0574; Cyp120; Cyp | Q59990 | Putative cytochrome P450 120 | -1,3 |
| Slr0335; ApcE | Q55544 | Phycobiliprotein | -1,4 |
| Smr0007; PsbL | Q55354 | Photosystem II reaction center protein L (PSII-L) | -1,6 |
| Sml0008; PsaJ | Q55329 | Photosystem I reaction center subunit IX | -1,7 |
| *Oxidative Phosphorylation* | | | |
| Sll1325; AtpH; AtpD | P27180 | ATP synthase d subunit | 1,5 |
| *Carbon Metabolism* |  |  |  |
| Ssl2501; PhaP | P73545 | Phasin (GA13) | 3,1 |
| Slr0009; CbbL; RbcL | P54205 | Ribulose bisphosphate carboxylase large chain (RuBisCO large subunit) | 1,6 |
| Slr0435; AccB | Q55120 | Biotin carboxyl carrier protein of acetyl-CoA carboxylase | 1,5 |
| Sll1841; OdhB | P74510 | Dihydrolipoamide acetyltransferase component of pyruvate dehydrogenase complex | 1,4 |
| Slr0233 | P52232 | Thioredoxin-like protein | 1,4 |
| Sll1070; TktA | P73282 | Transketolase (EC 2.2.1.1) | 1,4 |
| Slr1289; Icd | P80046 | isocitrate dehydrogenase (NADP+) | 1,3 |
| Slr1945; GpmI; Pgm | P74507 | 2,3-bisphosphoglycerate-independent phosphoglycerate mutase (iPGM) (EC 5.4.2.12) | 1,3 |
| Slr1994; PhaB | P73826 | Acetoacetyl-CoA reductase (EC 1.1.1.36) | 1,3 |
| Slr0752; Eno | P77972 | Enolase (EC 4.2.1.11) (2-phospho-D-glycerate hydro-lyase) (2-phosphoglycerate dehydratase) | 1,2 |
| Sll0990; FrmA | P73138 | S-(hydroxymethyl)glutathione dehydrogenase | 1,2 |
| Sll0861; MurQ | P73585 | N-acetylmuramic acid 6-phosphate etherase (MurNAc-6-P etherase) | -1,2 |
| Sll1383; SuhB | P74158 | Inositol-1-monophosphatase (I-1-Pase) (IMPase) (Inositol-1-phosphatase) (EC 3.1.3.25) | -1,4 |
| Slr1762 | P73039 | Phosphoglycolate phosphatase | -1,5 |
| *Cell Envelope and Lipid Metabolism* | | |  |
| Sll1951 | P73817 | S-layer protein (Hemolysin-like protein) (HLP) | 2,7 |
| *Cofactors and Vitamins Metabolism* | | |  |
| Sll1282; RibH | P73527 | 6,7-dimethyl-8-ribityllumazine synthase (DMRL synthase) (LS) (Lumazine synthase) | 1,8 |
| Sll1341; Bfr | P24602 | Bacterioferritin (BFR) (EC 1.16.3.1) | 1,6 |
| Slr1923 | P74473 | 3,8-divinyl protochlorophyllide a 8-vinyl-reductase | -1,2 |
| Slr1055; ChlH | P73020 | Mg-chelatase subunit ChlH (Anti-sigma factor E) | -1,3 |
| Sll0179; GltX | Q55778 | Glutamate--tRNA ligase (EC 6.1.1.17) (Glutamyl-tRNA synthetase) (GluRS) | -1,4 |
| Sll0250; CoaBC; Dfp | P73881 | Coenzyme A biosynthesis bifunctional protein CoaBC (DNA/pantothenate metabolism flavoprotein) (Phosphopantothenoylcysteine synthetase/decarboxylase) (PPCS-PPCDC) | -1,4 |
| Sll1184; PbsA1 | P72849 | Heme oxygenase 1 (EC 1.14.14.18) | -1,4 |
| Slr0506; Por; Pcr | Q59987 | Light-dependent protochlorophyllide reductase (PCR) (EC 1.3.1.33) (NADPH-protochlorophyllide oxidoreductase) (LPOR) (POR) | -1,5 |
| Slr1649; CpcT | P74371 | Chromophore lyase CpcT/CpeT | -1,5 |
| *Aminoacid Metabolism* | | |  |
| Slr0032; IlvE | P54691 | Probable branched-chain-amino-acid aminotransferase (BCAT) (EC 2.6.1.42) | 1,8 |
| Slr0229; MmsB | Q55702 | Uncharacterized oxidoreductase slr0229 | 1,5 |
| Slr1022; ArgD | P73133 | Acetylornithine aminotransferase (ACOAT) (EC 2.6.1.11) | 1,3 |
| Sll0585 | Q55865 | L-asparaginase | 1,3 |
| Sll1058; DapB | P72642 | 4-hydroxy-tetrahydrodipicolinate reductase (HTPA reductase) (EC 1.17.1.8) | 1,3 |
| Sll1750; UreC | P73061 | Urease subunit alpha (EC 3.5.1.5) (Urea amidohydrolase subunit alpha) | 1,3 |
| Sll0109; AroH | Q55869 | Chorismate mutase AroH (EC 5.4.99.5) | 1,2 |
| Sll1234; AhcY | P74008 | Adenosylhomocysteinase (EC 3.3.1.1) (S-adenosyl-L-homocysteine hydrolase) | 1,2 |
| Sll1987; KatG | P73911 | Catalase-peroxidase (CP) (EC 1.11.1.21) | 1,2 |
| Slr0662; SpeA1; SpeA | P74576 | Biosynthetic arginine decarboxylase 1 (ADC 1) (EC 4.1.1.19) | -1,2 |
| Slr1560; HisZ; HisS2 | P74592 | ATP phosphoribosyltransferase regulatory subunit | -1,2 |
| [Sll0712](https://www.genome.jp/dbget-bin/www_bget?syn:sll0712); CysM | P72662 | Cysteine synthase | -1,2 |
| Slr0738; TrpE | P20170 | Anthranilate synthase component 1 (AS) (ASI) (EC 4.1.3.27) | -1,2 |
| Slr1867; TrpD | P73617 | Anthranilate phosphoribosyltransferase (EC 2.4.2.18) | -1,3 |
| *Nucleotide Metabolism* | | |  |
| Sll0744 | Q55989 | dihydroorotate dehydrogenase (fumarate) | 1,3 |
| Slr1239; PntA | P73496 | Pyridine nucleotide transhydrogenase alpha subunit | 1,3 |
| Sll1815; Adk1 | P73302 | Adenylate kinase 1 (AK 1) (EC 2.7.4.3) | -1,4 |
| Slr1256; UreA | P73796 | Urease subunit gamma (EC 3.5.1.5) (Urea amidohydrolase subunit gamma) | -1,2 |
| Sll0509 | Q55478 | Ap-4-A phosphorylase II | -1,3 |
| *Transcription* |  |  |  |
| Slr8026 | Q6ZE66 | MarR family transcriptional regulatory protein | -1,3 |
| *Translation* |  |  |  |
| Sll1807; RplX; Rpl24 | P73309 | 50S ribosomal protein L24 | 1,7 |
| Ssl3437; RpsQ; Rps17 | P73311 | 30S ribosomal protein S17 | 1,5 |
| Sll1767; RpsF; Rps6 | P73636 | 30S ribosomal protein S6 | 1,5 |
| Sll1822; RpsI; Rps9 | P73293 | 30S ribosomal protein S9 | 1,4 |
| Sll1816; RpsM; Rps13 | P73299 | 30S ribosomal protein S13 | 1,3 |
| Sll1464; SelO | P73436 | Protein adenylyltransferase SelO (EC 2.7.7.-) | 1,4 |
| Slr0950 | P74319 | 23S rRNA (cytidine1920-2'-O)/16S rRNA (cytidine1409-2'-O)-methyltransferase | 1,3 |
| Sll1098; FusB; Fus | P74228 | Elongation factor G 2 (EF-G 2) | -1,2 |
| Sll1110; PrfA | P74707 | Peptide chain release factor 1 (RF-1) | -1,2 |
| Sll1253; PcnB | P74081 | A-adding tRNA nucleotidyltransferase (A-adding TNT) (EC 2.7.7.-) (A-adding enzyme) | -1,4 |
| *DNA Replication and Repair* | | |  |
| Slr1056 | P73021 | DNA replication and repair protein RecF | 1,4 |
| Sll0021; SbcD | Q55661 | Nuclease SbcCD subunit D | 1,4 |
| Slr0417; GyrA | Q55738 | DNA gyrase subunit A (EC 5.6.2.2) | -1,2 |
| Slr0833; DnaB | Q55418 | Replicative DNA helicase (EC 3.6.4.12) | -1,4 |
| *Protein / RNA Folding and Degradation* | | |  |
| Slr1377; LepB2 | P73157 | Probable signal peptidase I-2 (SPase I-2) (EC 3.4.21.89) (Leader peptidase I-2) | -1,5 |
| Slr0228; FtsH2 | Q55700 | FtsH Protease (quality control of Photosystem II in the thylakoid membrane) | -1,4 |
| *Motility* |  |  |  |
| [Slr0161](https://www.genome.jp/dbget-bin/www_bget?syn:slr0161); PilT | P74463 | Twitching motility protein | -1,3 |
| Slr1276; PilO | P74188 | Type IV pilus assembly protein | -1,7 |
| *Other Signalling and Cellular Processes* | | |  |
| Sll1957; ArsA | P73808 | Arsenical resistance operon repressor | 2,2 |
| Slr6037; ArsI2 | Q6YRW7 | Arsenate reductase - glutaredoxin-dependent family (use the GSH/glutaredoxin system) | 2,1 |
| Slr1198 | P73348 | Rehydrin | 1,8 |
| Sll0709; llaI.2 | P72665 | 2nd component required for LlaI restriction activity | 1,4 |
| Slr0242; Bcp | P72697 | Bacterioferritin comigratory protein | 1,3 |
| Slr1963 | P74102 | Orange carotenoid-binding protein (OCP) | 1,4 |
| Slr0088; CrtO | Q55808 | B-carotene ketolase | -1,3 |
| Slr1894 | P73321 | Starvation-inducible DNA-binding protein | -1,2 |
| Slr1205 | P73355 | Ferredoxin component | -1,4 |
| Slr0758; KaiC | P74646 | Circadian clock protein kinase KaiC (EC 2.7.11.1) | -1,4 |
| Sll0254 | P73872 | Carotenoid phi-ring synthase | -1,7 |
| *Transporters* |  |  |  |
| Sll1450; NrtA | P73452 | Nitrate Transport 45kDa protein | 1,9 |
| Sll1017 | P72935 | Putative Ammonium Transporter | 1,9 |
| Slr0530 | Q55472 | Membrane bound sugar transport protein | 1,7 |
| Sll1270; GlnH; GlnP | P73544 | Glutamine-binding periplasmic protein/glutamine transport system permease | 1,4 |
| Sll1180; HlyB | P74176 | ABC transporter | 1,2 |
| Sll0108 | P54147 | Putative ammonium transporter | 1,2 |
| Slr1881; LivF | P73650 | High-affinity branched-chain amino acid transport ATP-binding protein BraG | -1,2 |
| Sll1614; Pma1 | P37367 | Cation-transporting ATPase pma1 (EC 7.2.2.-) | -1,2 |
| Sll5052 | Q6ZES8 | Similar to Exopolysaccharide Export Protein | -1,3 |
| Slr0074; Ycf24 | Q55790 | ABC Transporter Subunit | -1,3 |
| Slr0625 | Q55868 | Glutamate: Na+ Symporter | -1,4 |
| *Unknown* |  |  |  |
| Slr1853 | P73604 |  | 2,8 |
| Ssl1046 | P74772 |  | 2,1 |
| Slr0168 | Q55549 |  | 2,1 |
| Sll0242 | P73896 |  | 2,0 |
| Ssr2406 | P73506 |  | 1,8 |
| Ssr2554 | P73961 |  | 1,7 |
| Sll5034 | Q6ZEU6 |  | 1,7 |
| [Slr1519](https://www.genome.jp/dbget-bin/www_bget?syn:slr1519); HglK | P73963 |  | 1,7 |
| Slr1752 | P73459 |  | 1,6 |
| Ssl2595 | P73587 |  | 1,6 |
| Ssr1407 | P74775 |  | 1,6 |
| Sll0781 | Q55953 |  | 1,6 |
| Sll1837 | P73107 | Periplasmic protein | 1,5 |
| Slr0545 | Q55493 | auxin-induced protein | 1,5 |
| Slr1618 | P72896 |  | 1,4 |
| Slr1576 | P74609 |  | 1,4 |
| Ssl0832 | P74691 |  | 1,4 |
| Sll1665 | P72805 |  | 1,4 |
| Ssl5113 | Q6ZEL7 |  | 1,4 |
| Slr1753 | P73032 |  | 1,4 |
| Slr1619 | P72897 |  | 1,4 |
| Slr0171; Ycf37 | Q55551 | Ycf37 gene product | 1,4 |
| Slr1194 | P73342 |  | 1,3 |
| Sll0737 | O06944 |  | 1,3 |
| Ssl2148 | P74239 |  | 1,3 |
| Sll1296 | P73172 | CheA like protein | 1,3 |
| Slr1437 | P73503 |  | 1,3 |
| Sll1150 | P73793 |  | 1,3 |
| Slr0650 | Q55730 |  | 1,3 |
| Slr1444 | P73516 |  | 1,2 |
| Slr6095 | Q6YRQ9 |  | -1,2 |
| Sll8004 | Q6ZE88 |  | -1,2 |
| Sll0487 | Q55818 |  | -1,2 |
| Sll0877 | P73552 |  | -1,2 |
| Slr1596; PcxA; CotA | P75028 | Proton extrusion protein PcxA | -1,3 |
| Sll1424 | P73944 |  | -1,3 |
| Sll0396 | Q55733 | OmpR subfamily | -1,3 |
| Sll8049 | Q6ZE43 | Type I site-specific deoxyribonuclease chain R | -1,3 |
| Sll1526 | P74360 |  | -1,3 |
| [Sll1636](https://www.genome.jp/dbget-bin/www_bget?syn:sll1636); Fbp | P73050 | Ferripyochelin binding protein | -1,3 |
| Sll2002 | P73680 |  | -1,3 |
| Sll0815 | P74042 |  | -1,3 |
| Slr1306 | P72844 |  | -1,3 |
| Sll1528 | P74358 |  | -1,3 |
| Slr1103 | P72747 |  | -1,3 |
| Sll0274 | P74392 |  | -1,3 |
| Slr0865 | P73759 |  | -1,3 |
| Sll0595 | Q55853 |  | -1,4 |
| Slr0399; Ycf39 | P74429 | Ycf39 gene product | -1,5 |
| Sll0529 | Q55517 |  | -1,5 |
| Slr2080 | P73905 |  | -1,5 |
| Slr5017 | Q6ZEW3 |  | -1,5 |
| Sll1730 | P73396 |  | -1,5 |
| Slr0937 | P74302 |  | -1,5 |
| Slr0320 | Q55524 |  | -1,5 |
| Slr6039 | Q6YRW5 |  | -1,6 |
| Sll1396 | P72619 |  | -1,8 |
| Ssl2733 | P72616 |  | -1,8 |
| Sll1891 | P74109 | Secreted protein (related to stress conditions) | -2,1 |
