# Supplementary material for: Absence of KpsM (Slr0977) Impairs the Secretion of Extracellular Polymeric Substances (EPS) and Impacts Carbon Fluxes in Synechocystis sp. PCC 6803
Source: mSphere. 2021 Jan 27;6(1):e00003-21. doi: 10.1128/mSphere.00003-21 (PMC7885315; doi:10.1128/mSphere.00003-21)
Supplement: TABLE S3 [file mSphere.00003-21-st003.docx]

**Table S3.** List of organisms and plasmids used/generated in this work.

| **Organism name/Genotype** | **Description** | **Source** |
| --- | --- | --- |
| *Escherichia coli* DH5α | Transformation/cloning strain. | Invitrogen |
| *Escherichia coli* XL1-Blue | Transformation/cloning strain. | Agilent |
| *Synechocystis* sp. PCC 6803 | Wild type strain. | PCC |
| *kpsM* mutant | *Synechocystis* mutant with *slr0977* replaced by a Km resistance cassette. | This work |
| *kpsM* mutant pS351slr0977 | *Synechocystis kpsM* mutant complemented with the replicative plasmid pS351slr0977. | This work |
| **Plasmid** | **Description** | **Source** |
| pGEM^®^-T easy | T/A cloning vector. | Promega |
| pSEVA351 | Replicative shuttle vector for *Synechocystis* transformation. | SEVA-DB [58] |
| pSEVA481 | Source of the Sm/Sp resistance cassette. | SEVA-DB |
| pKm.1 | pGEM-T easy with the Km resistance cassette. | [57] |
| pGDslr0977 | pGEM-T easy with *slr0977* and its flanking sequences, where the *slr0977* coding sequence was replaced by a *Xma*I site. | This work |
| pGDslr0977.Km | pGDslr0977 with a Km resistance cassette inserted into the *Xma*I site. | This work |
| pGDslr0977.Sm | pGDslr0977 with a Sm/Sp resistance cassette inserted into the *Sma*I site. | This work |
| pSBA2 | Source of the promoter of *psbA2** (P*_psbA2*_*) and the synthetic RBS BBa_B0030. | Registry of Standard Biological Parts (http://parts.igem.org). |
| pS351slr0977 | pSEVA351 with *slr0977* downstream the synthetic RBS BBa_B0030, under the control of P*_psbA2*_*. | This work |
