# Supplementary material for: Absence of KpsM (Slr0977) Impairs the Secretion of Extracellular Polymeric Substances (EPS) and Impacts Carbon Fluxes in Synechocystis sp. PCC 6803
Source: mSphere. 2021 Jan 27;6(1):e00003-21. doi: 10.1128/mSphere.00003-21 (PMC7885315; doi:10.1128/mSphere.00003-21)
Supplement: TABLE S4 [file mSphere.00003-21-st004.docx]

**Table S4.** Oligonucleotides used in this work.

| **Name** | **Sequence (5’-3’)** | **Purpose** | **Reference** |
| --- | --- | --- | --- |
| slr0977.5O | CGGATGCCACTATGCTTTTGAGTGATGAACC | Amplification of flanking region;  5I and 3I: overlap PCR | This work |
| slr0977.5I | cgttccatcttacgcccgggagaactgatta  ttgaagcaggacgcacgg* |  |  |
| slr0977.3I | tcaataatcagttctcccgggcgtaagatgg  aacgcaccttcgctgatgt* |  |  |
| slr0977.3O | GGATGGGGTCAGCCAGAAAATCTAACCAC |  |  |
| Slr0977Fwd_comp | GTTTCTTCGAATTCGCGGCCGCTTCTAGAGAT  GAAAACTTCCCCCCCAGA | Amplification of *kpsM* |  |
| Slr0977Rev_comp | GTTTCTTCCTGCAGCGGCCGCTACTAGTATTA  AATCACATCAGCGAAGGTGC |  |  |
| slr0978F_SB | CGTTGAGTGGAACCGTCGAAA | Southern Blot probe amplification |  |
| slr0980R_SB | CGGACTTCCTCCACTAAATTCTC |  |  |
| slr0977.5O.2 | CTTGGCATCCACCAGGGTCA | *kpsM* segregation confirmation |  |
| slr0977R | GTACCGCAATGTCCCGCCAA |  |  |
| KmRFwd | CCAGTCATAGCCGAATAGCCTC |  |  |
| KmRRev | gcatcgccttctatcgcctt |  |  |
| Km.KmScFwd | CTGACCCCGGGTGAATGTCAGCTACTGG* | Amplification of the Km resistance cassette | [57] |
| KmRev | CAAACCCGGGCGATTTACTTTTCGACCTC* |  |  |

*Underlined base pairs correspond to restriction sites.
