# Supplementary material for: Absence of KpsM (Slr0977) Impairs the Secretion of Extracellular Polymeric Substances (EPS) and Impacts Carbon Fluxes in Synechocystis sp. PCC 6803
Source: mSphere. 2021 Jan 27;6(1):e00003-21. doi: 10.1128/mSphere.00003-21 (PMC7885315; doi:10.1128/mSphere.00003-21)
Supplement: TEXT S1 [file mSphere.00003-21-s0001.docx]

**Supporting Information**

**Supporting Experimental Procedures**

***Detailed description of the RNA Sequencing experiment***

The trancriptomes of *Synechocystis* wild type and *kpsM* mutant were analyzed by RNA sequencing, using three biological replicates. Cultures were grown in the conditions described at the Experimental procedures section until reaching an OD_730nm_ of 1.5. At that point, 100 ml of each culture was centrifuged at 3850 g for 5 min at room temperature, and cells were washed with sterile BG11 and pre-treated with RNAprotect Bacteria Reagent® (Qiagen) before saved at -80 ºC. After RNA extraction, a total amount of 1 µg RNA per sample was used as input material for the RNA sample preparations. Sequencing libraries were generated using NEBNext® Ultra TM RNA Library Prep Kit for Illumina® (NEB, USA) following manufacturer’s recommendations and index codes were added to attribute sequences to each sample. Briefly, mRNA was purified from total RNA using poly-T oligo-attached magnetic beads. Fragmentation was carried out using divalent cations under elevated temperature in NEBNext First Strand Synthesis Reaction Buffer (5X). First strand cDNA was synthesized using random hexamer primer and M-MuLV Reverse Transcriptase (RNase H-). Second strand cDNA synthesis was subsequently performed using DNA Polymerase I and RNase H. Remaining overhangs were converted into blunt ends via exonuclease/polymerase activities. After adenylation of 3’ ends of DNA fragments, NEBNext Adaptor with hairpin loop structure were ligated to prepare for hybridization. In order to select cDNA fragments of preferentially 150~200 bp in length, the library fragments were purified with AMPure XP system (Beckman Coulter, Beverly, USA). Then 3 µl USER Enzyme (NEB, USA) was used with size-selected, adaptorligated cDNA at 37 °C for 15 min followed by 5 min at 95 °C before PCR. Then PCR was performed with Phusion High-Fidelity DNA polymerase, Universal PCR primers and Index (X) Primer. At last, PCR products were purified (AMPure XP system) and library quality was assessed on the Agilent Bioanalyzer 2100 system. The clustering of the index-coded samples was performed on a cBot Cluster Generation System using PE Cluster Kit cBot-HS (Illumina) according to the manufacturer’s instructions. After cluster generation, the library preparations were sequenced on an Illumina platform and paired-end reads were generated. Raw data (raw reads) of FASTQ format were firstly processed through in-house scripts. In this step, clean data (clean reads) were obtained by removing reads containing adapter and poly-N sequences and reads with low quality from raw data. At the same time, Q20, Q30 and GC content of the clean data were calculated. All the downstream analyses were based on the clean data with high quality. Reference genome and gene model annotation files were downloaded from genome website browser (NCBI/UCSC/Ensembl) directly. Paired-end clean reads were mapped to the reference genome using HISAT2 software. HISAT2 uses a large set of small GFM indexes that collectively cover the whole genome. These small indexes (called local indexes), combined with several alignment strategies, enable rapid and accurate alignment of sequencing reads. HTSeq was used to count the read numbers mapped of each gene, including known and novel genes. And then RPKM of each gene was calculated based on the length of the gene and reads count mapped to this gene. RPKM, Reads Per Kilobase of exon model per Million mapped reads, considers the effect of sequencing depth and gene length for the reads count at the same time, and is currently the most commonly used method for estimating gene expression levels. Differential expression analysis between two conditions/groups (three biological replicates per condition) was performed using DESeq2 R package. DESeq2 provides statistical routines for determining differential expression in digital gene expression data using a model based on the negative binomial distribution. The resulting P values were adjusted using the Benjamini and Hochberg’s approach for controlling the False Discovery Rate (FDR). Genes with an adjusted P value < 0.05 found by DESeq2 were assigned as differentially expressed.

***Detailed description of iTRAQ experiment***

The proteomes of Synechocystis wild type and *kpsM* mutant were analyzed by 8-plex isobaric tags for relative and absolute quantification (iTRAQ), using three biological replicates. Cultures were grown in the conditions described at the Experimental procedures section until reaching a OD_730nm_ of 1.5. At that point, 75 ml of each culture was centrifuged at 3850 g for 5 min at room temperature, and cells were washed with K phosphate buffer (50 mM K2HPO4, 50 mM KH2PO4, pH 6.9) before saved at -80 ºC. Cell pellets of WT (3 biological replicates) and *kpsM* mutant (3 biological replicates), were thawed and cells were re-suspended in 400 μL lysis buffer [200 mM triethylammonium bicarbonate (TEAB), 10 mM dithiothreitol (DTT), 1% (w/v) sodium deoxycholate, 0.1% (v/v) NP40 and 5 μL protease inhibitor cocktail set II, pH 8.5] and transferred to 2 mL LoBind microcentrifuge tubes (Eppendorf) containing ∼400 mg of 200 µm Zirconium beads. Cell lysis was performed using a cell disruptor (Genie, VWR, UK) using 10 cycles of 60 s with a one minute cooling step on ice between cycles. Samples were centrifuged at 13,000 g for 10 min at 4°C, and supernatants were transferred to clean microcentrifuge tubes. Remaining pellets were further resuspended in 200 µL of lysis buffer, and a further 5 cycles on the cell disruptor was used as before. Samples were centrifuged and supernatants were combined. The combined supernatants were further clarified by centrifugation at 13,000 g for 10 min at 4°C to ensure that any remaining cell debris was removed from the extracts. The clarified extracts were incubated with 2 µl of benzonase nuclease (Novagene) for 2 min on ice. Protein content in each extract was estimated using the modified Lowry spectrophotometric method as previously described [1]. The concentration of the protein extracts was evaluated by sodium dodecyl sulfate polyacrylamide gel [2] followed by silver staining [3]. Isobaric tags for relative and absolute quantitation (iTRAQ) 8-plex labelling was performed according to the manufacturer’s protocol (8-plex iTRAQ reagent Multiplex kit, ABSciex, USA). Before labelling, samples were reduced, alkylated and digested as follows: 100 µg of proteins from each sample was first reduced with 2.5 µL of 10 mM Tris (2-carboxyethyl) phosphine hydrochloride and incubated at 60 °C for 1 h. Samples were then alkylated using 4.5 μL of 20 mM methyl methanethiosulfonate at room temperature for 30 min. Subsequently, proteins were digested with trypsin (Promega, UK) at a ratio of 1:20 (trypsin:protein) overnight at 37 °C. After digestion, biological replicates from each Synechocystis strain (WT and *kpsM* mutant) were labelled with a specific iTRAQ reagent. Labels 113, 114 and 115 were used to label the WT samples and labels 116, 117 and 118 were used to label the *kpsM* mutant samples. After 2 h incubation at room temperature, iTRAQ-labelled peptides were combined and concentrated using a vacuum concentrator (Eppendorf). iTRAQ-labelled peptides were off-line fractionated using a porous graphitic column (Hypercarb) with the following specifications: 7 μm particle size, 50 mm length, 2.1 mm diameter and 250 Å pore size (Thermo Fisher Scientific, Waltham, MA, USA) coupled with an UHPLC Ultimate 3000 RS (Thermo Fisher Scientific, Dionex, Hemel Hempstead, UK) at a flow rate of 0.2 mL min^−1^. iTRAQ-labelled peptides were resuspended in 100 μL of buffer A [97% (v/v) HPLC water, 3% (v/v) HPLC acetonitrile, 0.1% (v/v) trifluoroacetic acid] and separated using a 86 minutes gradient starting with 2% buffer B [97% (v/v) HPLC acetonitrile, 3% (v/v) HPLC water, 0.1% (v/v) trifluoroacetic acid] for 5 min, 2–10% buffer B for 5 min, 10–60% buffer B for 50 min, 60–80% buffer B for 10 min, 80–90% buffer B for 1 min, 90% buffer B for 5 min and 90-2% buffer B for 1 min and 2% buffer B for 9 min. The chromatographic profile of the separated peptides was monitored at the wavelength of 240 nm using Chromeleon software (Thermo Fisher Scientific, Hemel, Hempstead, UK). Fractions were collected every 2 min from 10 min to 50 min (20 fractions). Collected fractions were then dried in a vacuum concentrator and stored at −20 °C until further analysis.

Each fraction was resuspended in 10 μL reverse phase (RP) buffer A [97% (v/v) HPLC water, 3% (v/v) HPLC acetonitrile, 0.1% (v/v) formic acid] and combined to obtain 10 fractions for mass spectrometric analysis. Each fraction was run using a Q Exactive^TM^ Hybrid Quadrupole-Orbitrap^TM^ mass spectrometer (Thermo Scientific, Bremen, Germany) coupled with an online UHPLC Ultimate 3000 (Thermo Fisher Scientific, Dionex, Hemel Hempstead, UK). From each fraction, 4 μL were injected and peptides were separated using a PepMap RSLC C18 column with the following characteristics: 2 μm, 100 Å, 75 μm × 50 cm (Thermo Fisher Scientific, Hemel, Hempstead, UK) at a constant flow rate of 300 nL min^−1^. A 135 min gradient was performed using RP buffer B [97% (v/v) HPLC acetonitrile, 3% (v/v) HPLC water, 0.1% (v/v) formic acid] as follows: 4% B for 0 min, 4% B for 5 min, 4–40% B for 100 min, 40–90% B for 1 min, 90% B for 14 min, 90–4% for 1 min and finally 4% of buffer B for 14 min. Mass spectrometry (MS) data was acquired using Xcalibur software v 4.0 (Thermo Scientific, Bremen, Germany) with the following settings. MS scans were acquired with 60,000 resolution, automatic gain control (AGC) target 3e6, maximum injection time (IT) 100 ms. The MS mass range was set to be in the range 100–1500 m/z. Tandem mass spectrometry (MS/MS) scans were acquired using high-energy collision dissociation (HCD), 30,000 resolution, AGC target 5e4, maximum IT 120 ms. In total, 15 MS/MS were acquired per MS scan using normalised collision energy (NCE) of 34% and isolation window of 1.2 m/z. The *Synechocystis* sp. PCC 6803 (taxon ID: 1111708) database containing 3507 proteins was downloaded from Uniprot (.fasta) and uploaded on MaxQuant software (version 1.5.4.1). The settings were as follows; for “type the experimental set” MS2 and 8-plex iTRAQ were selected with reporter mass tolerance of 0.01 Da. Enzymatic digestion with trypsin was specified and two missed cleavages were allowed per peptide. Oxidation of methionine and deamidation of asparagine and glutamine were selected as variable modifications and methylthio modification of cysteine was selected as the fixed modification. The false discovery rate (FDR) at the peptide spectrum match/protein level was set at 1%. The reporter ions intensities (113, 114, 115, 116, 117 and 118) were used for quantification purposes. Fold changes of the differentially abundant proteins were calculated using a published method [4] and using the ProteoSign, an online service.

***References***

[1] Bensadoun, A., and Weinstein, D. (1976) Assay of proteins in the presence of interfering materials. *Analytical Biochemistry* 70: 241−250.

[2] Laemmli, U.K. (1970) Cleavage of structural proteins during the assembly of the head of bacteriophage T4. *Nature* 227: 680–685.

[3] Couto, N., Barber, J., and Gaskell, S.J. (2011) Matrix-assisted laser desorption/ionization (MALDI) mass spectrometric response factors of peptides generated using different proteolytic enzymes. *Journal of Mass Spectrometry* 46: 1233−1240.

[4] Efstathiou, G., Antonakis, A.N., Pavlopoulos, G.A., Theodosiou, T., Divanach, P., Trudgian, D.C., Thomas, B., Papanikolaou, N., Aivaliotis, M., Acuto, O., and Iliopoulos, I. (2017) ProteoSign: an end-user online differential proteomics statistical analysis platform. *Nucleic Acids Research* 45: W300–W306.

[5] Trautner, C., and Vermaas, W.F.J. (2013) The *sll1951* gene encodes the surface layer protein of *Synechocystis* sp. Strain PCC 6803. *Journal of Bacteriology* 195: 5370–5380.
